# Tobacco smoking and the risk of abdominal aortic aneurysm: a systematic review and meta-analysis of prospective studies

**DOI:** 10.1038/s41598-018-32100-2

**Published:** 2018-10-03

**Authors:** Dagfinn Aune, Sabrina Schlesinger, Teresa Norat, Elio Riboli

**Affiliations:** 1 Department of Epidemiology and Biostatistics, School of Public Health, Imperial College London, London, United Kingdom; 2 Department of Endocrinology, Morbid Obesity and Preventive Medicine, Oslo University Hospital, Oslo, Norway; 3 Department of Nutrition, Bjørknes University College, Oslo, Norway; 4 Institute for Biometry and Epidemiology, German Diabetes Center, Leibniz Institute for Diabetes Research at the Heinrich-Heine-University Düsseldorf, Düsseldorf, Germany

## Abstract

Several studies have found that smoking increases the risk of abdominal aortic aneurysm, however, the strength of the association has differed between studies and data from cohort studies have not yet been summarized. A systematic review and meta-analysis was therefore conducted to clarify this association. We searched PubMed and Embase databases up to May 2^nd^ 2018. A random effects model was used to estimate summary relative risks (RRs) and 95% confidence intervals (CIs). Twenty three prospective studies were included. Comparing current, former and ever smokers with never smokers the summary RRs were 4.87 (95% CI: 3.93–6.02, I^2^ = 92%, n = 20), 2.10 (95% CI: 1.76–2.50, I^2^ = 71%, n = 15) and 3.28 (95% CI: 2.60–4.15, I^2^ = 96%, n = 18), respectively. The summary RR was 1.87 (95% CI: 1.45–2.40, I^2^ = 97%) per 10 cigarettes per day, 1.78 (95% CI: 1.54–2.06, I^2^ = 83%) per 10 pack-years was and 0.45 (95% CI: 0.32–0.63, I^2^ = 92.3%) per 10 years of smoking cessation. There was evidence of nonlinearity for cigarettes per day and pack-years (p_nonlinearity_ < 0.0001 and p_nonlinearity_ = 0.02, respectively), but not for smoking cessation, p_nonlinearity_ = 0.85. Among smokers who quit, the RR was similar to that of never smokers by 25 years of smoking cessation. These findings confirm a strong association between smoking and the risk of developing abdominal aortic aneurysms.

## Introduction

Aortic aneurysms are localized dilatations of the aorta that exceeds the normal diameter by 50% or >3 cm^1^. Rupture of an aortic aneurysm can cause massive internal bleeding and is usually fatal; 80% of those reaching hospital and 50% of those undergoing surgery for ruptured aortic aneurysms die as a consequence^2,3^. Globally aortic aneurysms accounted for 168200 deaths and 2.9 million disability-adjusted life years in 2015^4,5^. Most aortic aneurysms are abdominal aortic aneurysms, and these are more common in men than in women^6^, although the reason for these sex differences are unclear. The incidence of new abdominal aortic aneurysms is 0.4–0.67% in Western populations^7–9^, and ten-fold lower in Asian populations^10^. Secular trend studies of abdominal aortic aneurysms have shown conflicting results. An early American study found increased incidence of abdominal aortic aneurysms between 1951 and 1980^11^ and a Swedish study found an approximate doubling in the incidence rate of ruptured abdominal aortic aneurysms between 1971–1986 and 2000–2004 in spite of increased use of surgical treatment^12^. However, another study found an increased incidence of non-ruptured aneurysms between 1990 and 2005, but no change in the incidence of interventions for ruptured aneurysms^13^, while a third study found a lower prevalence of abdominal aortic aneurysm detected through ultrasound screening over time which the authors attributed to a lower prevalence of smoking in more recent decades^14^. A study from Finland found a reduced incidence and mortality from ruptured abdominal aortic aneurysms between 2003 and 2013^15^. Some of the established risk factors include age, ethnicity, height, hypertension, coronary heart disease, peripheral artery disease, while diabetes appears to be associated with a reduced risk^16^.

Although a large number of studies have found a positive association between smoking and risk of abdominal aortic aneurysm^7,16–36^, there has been variation observed with regard to the strength of the association between studies and by sex, with stronger associations observed in women than in men in some^30,33^, although not all studies^22,32,34^. Several studies found a dose-response relationship between the intensity of smoking and risk of abdominal aortic aneurysms^7,18,28,29^, however, in a few studies there was a flattening of the curve with a larger number of cigarettes smoked per day^20,22,25,27^. More recently, studies of smoking cessation have quite consistently reported reduced risk with longer duration of smoking cessation^7,26,30,34^, however, while some studies suggested a risk similar as in never smokers with very long durations of smoking cessation^7,30^, other studies found some elevation in risk even after long durations of smoking cessation^26,30,34^. Although a previous meta-analysis found an increased risk of abdominal aortic aneurysms among smokers, only screening studies (cross-sectional studies) were included in that analysis^37^. As cross-sectional studies cannot be used to draw causal inferences we therefore conducted a systematic review and meta-analysis of prospective studies on the association between tobacco smoking and the risk of abdominal aortic aneurysms to provide better evidence from studies with a stronger study design. We aimed to clarify the strength and shape of the dose-response relationship between tobacco smoking and risk of abdominal aortic aneurysm, potential differences of the association by sex, and the effects of smoking cessation.

## Material and Methods

### Search strategy

We searched the databases Pubmed, and Embase up to May 2^nd^ 2018 for eligible studies (DA, SS). The search terms used for the searches are found in the Supplementary Text. We also screened the reference lists of the identified publications for additional potentially relevant studies. We followed the MOOSE criteria with regard to reporting of meta-analyses^38^.

### Study selection

Published retrospective and prospective cohort studies and nested case-control studies within cohort studies on tobacco smoking and the risk of abdominal aortic aneurysms were eligible for inclusion. For studies to be included adjusted relative risk (RR) estimates had to be published with the 95% confidence intervals (CIs). A list of the excluded studies and the reasons for exclusion are found in Supplementary Table 1.

### Data extraction

We extracted the following data from each study: The first author’s last name, publication year, country where the study was conducted, study period, sample size, number of cases and participants, subgroup, relative risks and 95% CIs for smoking exposure and the confounders adjusted for in the analysis. The data extraction was done by DA and checked for accuracy by SS.

### Statistical methods

We calculated summary RRs and 95% CIs of abdominal aortic aneurysm by smoking status, cigarettes per day, pack-years, and time since quitting smoking using random-effects models^39^ which take into account heterogeneity between studies. A weighted average of the natural logarithm of the RRs was estimated using random effects weights^39^. The main analysis included studies that analyzed current, former, or ever smokers vs. never smokers (which was used as the reference category). As there is evidence that even former smokers are at increased risk, we conducted a separate analysis of studies that analyzed current vs. non-current smokers (former + never smokers). Linear dose-response analyses were conducted using the method of Greenland and Longnecker^40^. Study-specific linear trends and 95% CIs were computed from the natural log of the RRs and CIs across categories of cigarettes per day, pack-years and years since smoking cessation. A potential nonlinear association was investigated using restricted cubic splines with three knots at 10%, 50%, and 90% percentiles of the distribution, which was combined using multivariate meta-analysis^41,42^. For one study^23^ we used a previously described method to estimate approximate confidence intervals based on the size of the hazard ratios and the distribution of the participants for each smoking category^43^.

Q and I^2^ statistics were used to assess heterogeneity between studies^44^. I^2^ is a measure of the amount of heterogeneity that is due to between study variation rather than chance. Subgroup analyses stratified by duration of follow-up, outcome type, sex, geographic location, number of cases, study quality and adjustment for confounding factors were conducted to investigate potential sources of heterogeneity. The Newcastle Ottawa scale was used to assess study quality and it rates studies according to selection, comparability and outcome assessment with a score range from 0 to 9^45^. Egger’s test^46^, Begg-Mazumdar’s test^47^ and inspection of funnel plots were used to assess publication bias. Stata 13.0 software (StataCorp, Texas, US) was used for the statistical analyses.

## Results

**Figure 1:**
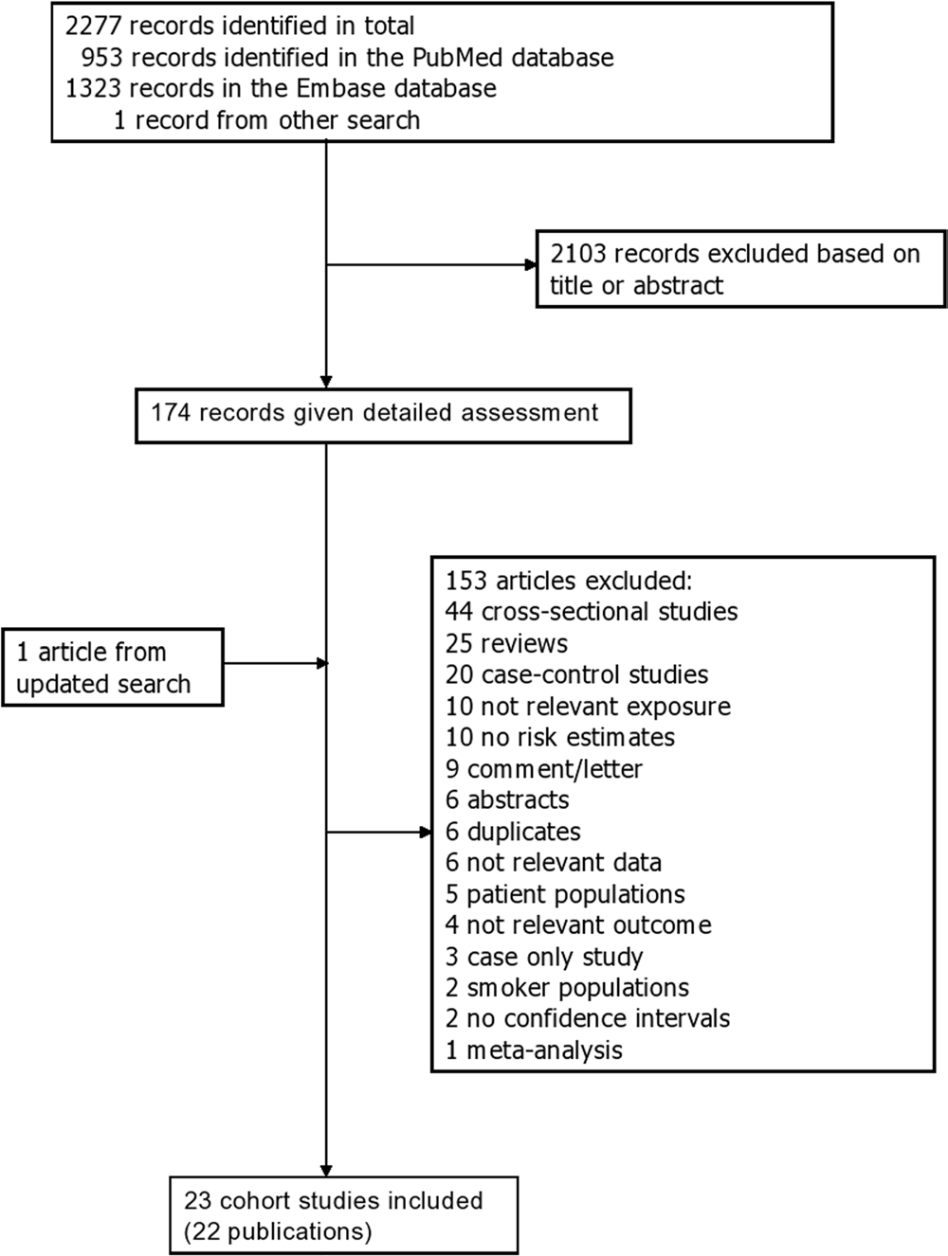
Flow-chart of study selection.

Twenty three prospective studies (22 publications)^7,16–36^ were included in the meta-analysis of smoking and risk of abdominal aortic aneurysms (Fig. 1, Supplementary Table 2). Thirteen studies were from Europe, eight studies were from the USA, and two studies were from Asia (Supplementary Table 2). The study characteristics of the studies included in the meta-analysis are provided in Supplementary Table 2 including study period, number of cases and participants, age, comparisons and risk estimates and confounders adjusted for.

### Smoking status

**Figure 2:**
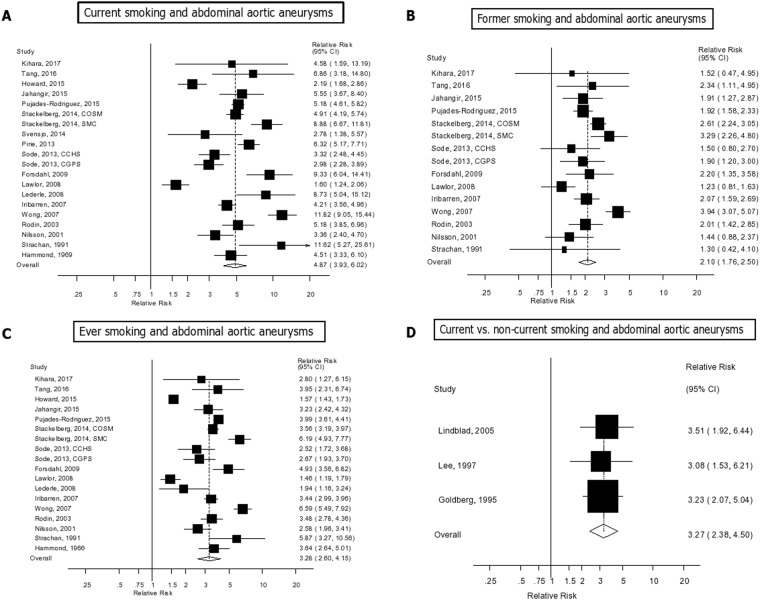
Smoking status and abdominal aortic aneurysm.

Twenty studies (18 publications)^7,16,18,19,22,23,25–36^ of current smokers (8901 cases, 4716185 participants), fifteen studies (thirteen publications)^7,19,22,23,25–27,29–31,33,35,36^ of former smokers (7824 cases, 3060503 participants), and eighteen studies (16 publications)^7,16,17,19,22,23,25–27,29,30,32–36^ of ever smokers (8448 cases, 3934635 participants) and abdominal aortic aneurysms were included in the analyses. The summary RR for current smokers vs. never smokers was 4.87 (95% CIs: 3.93–6.02, I^2^ = 91.5%, p_heterogeneity_ < 0.0001) (Fig. 2A), for former smokers it was 2.10 (95% CI: 1.76–2.50, I^2^ = 71.3%, p_heterogeneity_ < 0.0001) (Fig. 2B), and for ever smokers it was 3.28 (95% CI: 2.60–4.15, I^2^ = 95.8%, p_heterogeneity_ < 0.0001) (Fig. 2C). There was no evidence of publication bias with Egger’s test, p = 0.86 for current smokers, 0.18 for former smokers, and 0.48 for ever smokers, respectively (Supplementary Figs 1–3). In three studies^20,21,24^ of current smokers vs. never/former smokers combined the summary RR was 3.27 (95% CI: 2.38–4.50, I^2^ = 0%, p_heterogeneity_ = 0.96) (Supplementary Fig. 2d).

### Cigarettes per day, pack-years and years since quitting smoking

**Figure 3:**
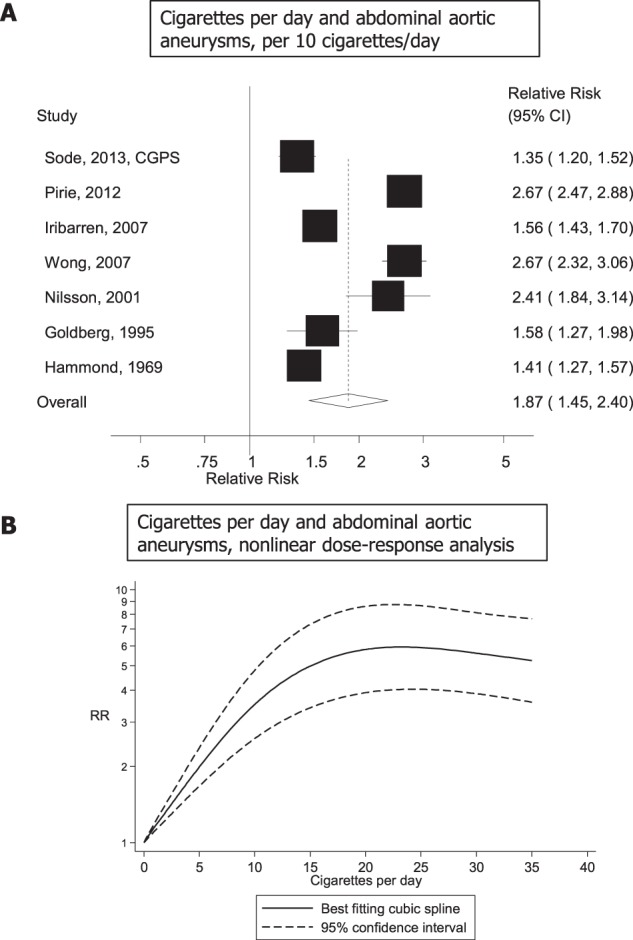
Cigarettes per day and abdominal aortic aneurysm, linear and nonlinear dose-response analysis.

Seven studies^18,20,22,25,26,28,29^ analysed the association between number of cigarettes per day and abdominal aortic aneurysms (2219 cases, 1658103 participants) and the summary RR per 10 cigarettes per day was 1.87 (95% CI: 1.45–2.40, I^2^ = 96.7%, p_heterogeneity_ < 0.0001) (Fig. 3A). There was evidence of a nonlinear association between number of cigarettes per day and abdominal aortic aneurysms, p_nonlinearity_ < 0.0001, with a strong increase in risk up to 15–20 cigarettes per day, but no further increase beyond 20 cigarettes per day (Fig. 3B). The summary RRs for 5, 10, 15, 20 and 25 cigarettes per day compared to zero 1.99 (1.67–2.37), 3.52 (2.58–4.80), 4.99 (3.41–7.29), 5.81 (3.92–8.60), 5.91 (4.04–8.64), and 5.61 (3.88–8.11), respectively (Fig. 3B, Supplementary Table 3).

**Figure 4:**
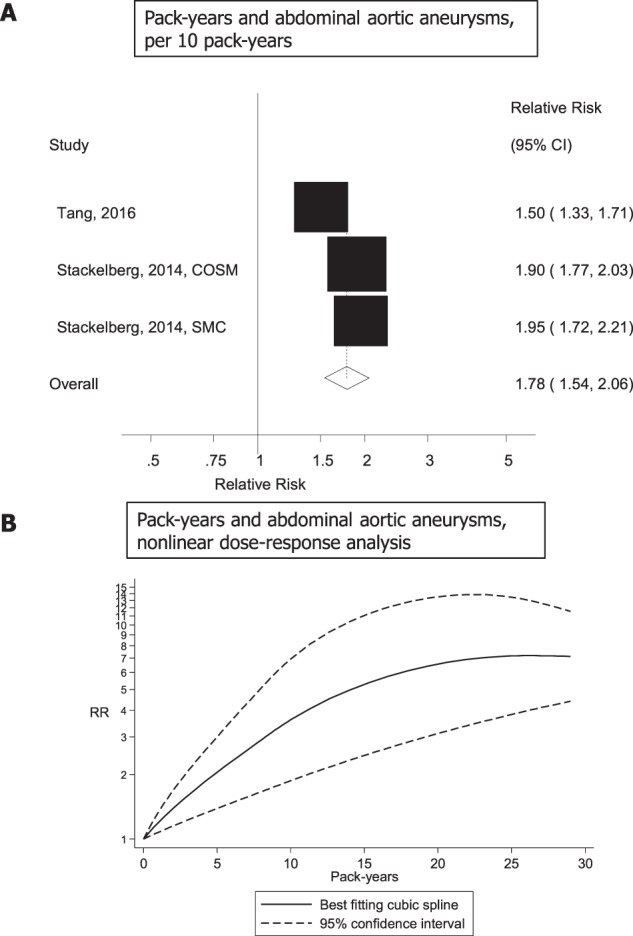
Pack-years and abdominal aortic aneurysm, linear and nonlinear dose-response analysis.

Three studies (two publications)^30,35^ on the association between number of pack-years of smoking and abdominal aortic aneurysms (1747 cases, 93938 participants) were included and the summary RR was 1.78 (95% CI: 1.54–2.06, I^2^ = 82.5%, p_heterogeneity_ < 0.0001) per 10 pack-years (Fig. 4A). There was also evidence of nonlinearity in the analysis of pack-years of smoking and abdominal aortic aneurysms, p_nonlinearity_ = 0.02, with a strong increase in risk up to 20–25 pack-years, but little further increase in risk above 25 pack-years (Fig. 4B), however, there were few data points at higher levels of pack-years. The summary RRs for 5, 10, 15, 20, 25 and 30 pack-years compared to zero pack-years were 2.01 (1.38–2.92), 3.60 (1.87–6.94), 5.34 (2.46–11.63), 6.61 (3.11–14.11), 7.17 (3.81–13.45), and 7.08 (4.55–11.01), respectively (Fig. 4B, Supplementary Table 4).

**Figure 5:**
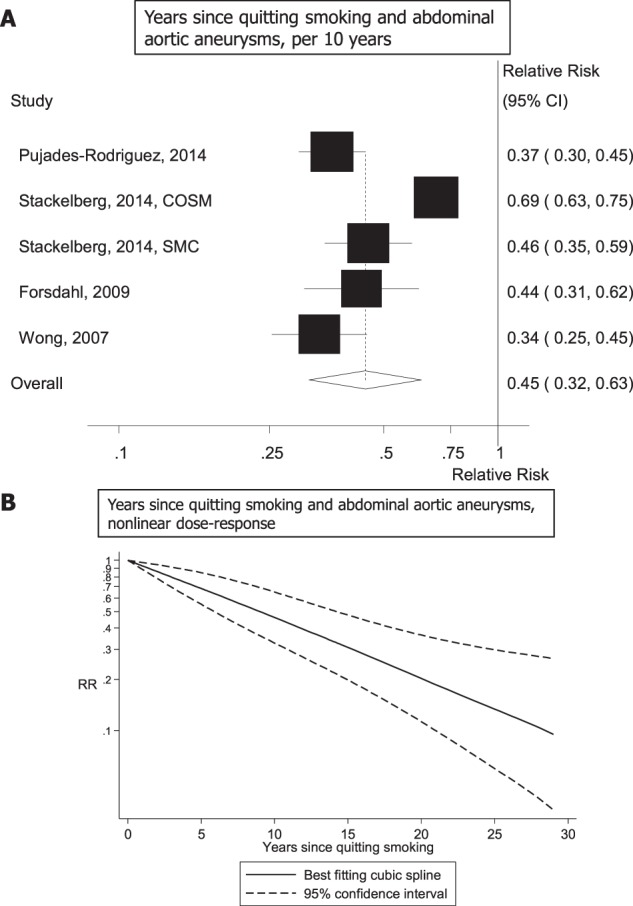
Years since quitting smoking and abdominal aortic aneurysm, linear and nonlinear dose-response analysis.

Five studies (4 publications)^7,26,30,34^ investigating the number of years since quitting smoking and risk of abdominal aneurysms (4787 cases, 2059203 participants) were included and the summary RR per 10 years of smoking cessation was 0.45 (95% CI: 0.32–0.63, I^2^ = 92.3%, p_heterogeneity_ < 0.0001) (Fig. 5A). There was no evidence of nonlinearity, p_nonlinearity_ = 0.85, and the relative risk at 25 years since quitting, summary RR = 0.13 (95% CI: 0.06–0.30), was similar to that of never smokers (summary RR = 0.14, 95% CI: 0.10–0.19) (Fig. 5B, Supplementary Table 5).

### Subgroup and sensitivity analyses

Positive associations were observed between current, former and ever smoking and risk of abdominal aortic aneurysms across nearly all subgroups defined by duration of follow-up, outcome type, sex, geographic location, number of cases, study quality and adjustment for confounding factors (including age, education, alcohol, height, BMI, and physical activity, cardiovascular disease, hypertension, hypercholesterolemia, serum cholesterol, and diabetes) (Table 1). There was no evidence that the results differed between these subgroups in meta-regression analyses, except for two subgroup analyses stratified by adjustment for hypertension and hypercholesterolemia among former smokers, where there was a stronger association among studies with such adjustment compared to studies without such adjustment (p_heterogeneity_ = 0.04 for both). The association between current and ever smoking versus never smoking and abdominal aortic aneurysms appeared to be slightly stronger among women than among men, although the test for heterogeneity between subgroups was not significant, p_heterogeneity_ = 0.44 and p_heterogeneity_ = 0.75, respectively.

**Table 1:** Subgroup analyses of smoking status and abdominal aortic aneurysms.

		Current Smoking					Former smoking					Ever smoking				
		*n*	RR (95% CI)	*I*^2^ (%)	*P*_h_^1^	*P*_h_^2^	*n*	RR (95% CI)	*I*^*2*^ (%)	*P*_h_^1^	*P*_h_^2^	*n*	RR (95% CI)	*I*^*2*^ (%)	*P*_h_^1^	*P*_h_^2^
All studies		20	4.87 (3.93–6.02)	91.5	<0.0001		15	2.10 (1.76–2.50)	71.3	<0.0001		18	3.28 (2.60–4.15)	95.8	<0.0001	
Follow-up																
<10 years		7	5.06 (3.84–6.65)	80.0	<0.0001	0.82	4	1.94 (1.66–2.27)	0	0.96	0.69	6	3.43 (2.81–4.19)	68.2	0.008	0.94
≥10 years		13	4.79 (3.52–6.51)	93.7	<0.0001		11	2.12 (1.67–2.70)	76.8	<0.0001		12	3.28 (2.37–4.53)	96.9	<0.0001	
Outcome type																
Incidence		16	4.94 (3.84–6.36)	92.9	<0.0001	0.82	13	2.30 (2.12–2.50)	73.3	<0.0001	0.26	16	3.02 (2.89–3.16)	96.3	<0.0001	0.99
Mortality		4	4.67 (3.33–6.53)	72.8	0.01		2	1.45 (0.92–2.29)	0	0.93		2	3.51 (2.61–4.72)	0	0.55	
Sex																
Men		9	4.55 (4.19–4.94)	94.8	<0.0001	0.63/0.44^3^	6	2.43 (2.17–2.73)	88.2	<0.0001	0.94/0.44^3^	8	2.57 (2.42–2.73)	97.2	<0.0001	0.47/0.75^3^
Women		7	5.79 (5.14–6.53)	83.1	<0.0001		3	3.14 (2.29–4.32)	52.1	0.12		6	3.41 (2.95–3.95)	93.5	<0.0001	
Men and women		8	4.24 (3.82–4.70)	72.5	0.001		7	2.00 (1.70–2.36)	0	0.96		8	3.69 (3.44–3.96)	52.7	0.04	
Geographic location																
Europe		11	4.68 (3.67–5.97)	89.9	<0.0001	0.60	8	2.12 (1.74–2.58)	57.0	0.02	0.49	9	3.40 (2.41–4.80)	97.1	<0.0001	0.32
America		7	6.17 (4.39–8.67)	87.1	<0.0001		5	2.40 (1.72–3.35)	77.5	0.001		7	3.66 (2.80–4.77)	86.5	<0.0001	
Asia		2	2.38 (0.88–6.45)	72.1	0.06		2	1.25 (0.89–1.75)	0	0.74		2	1.80 (0.99–3.27)	59.3	0.12	
Number of cases																
<250		9	5.01 (3.21–7.81)	90.6	<0.0001	0.99	6	2.06 (1.51–2.80)	43.0	0.12	0.73	8	3.17 (1.96–5.14)	95.8	<0.0001	0.60
250–<500		7	4.68 (2.72–8.03)	95.4	<0.0001		5	1.98 (1.24–3.18)	88.0	<0.0001		6	2.92 (1.65–5.16)	95.8	<0.0001	
≥500		4	4.84 (4.32–5.42)	37.7	0.19		4	2.22 (1.85–2.66)	53.7	0.09		4	3.70 (3.43–3.99)	19.8	0.29	
Study quality																
0–3 stars		1	11.62 (5.27–25.61)			0.70	1	1.30 (0.42–4.10)			0.25	1	5.87 (3.27–10.56)			0.83
4–6 stars		6	4.71 (2.89–7.66)	95.4	<0.0001		3	1.88 (1.60–2.21)	0	0.52		6	3.16 (1.93–5.19)	95.8	<0.0001	
7–9 stars		14	4.92 (3.63–6.66)	93.1	<0.0001		11	2.21 (1.77–2.76)	74.5	<0.0001		11	3.50 (2.67–4.58)	93.4	<0.0001	
Adjustment for confounding factors^3^																
Age	Yes	19	4.72 (3.81–5.86)	91.7	<0.0001	0.19	14	2.12 (1.77–2.53)	72.8	<0.0001	0.47	17	3.20 (2.52–4.06)	96.0	<0.0001	0.26
	No	1	11.62 (5.27–25.62)				1	1.30 (0.42–4.10)				1	5.87 (3.27–10.56)			
Education	Yes	5	5.48 (4.16–7.22)	80.0	<0.0001	0.60	5	2.41 (2.00–2.90)	39.7	0.16	0.35	5	3.85 (3.09–4.81)	82.2	<0.0001	0.41
	No	15	4.71 (3.54–6.26)	93.0	<0.0001		10	1.94 (1.48–2.53)	76.6	<0.0001		13	3.12 (2.27–4.30)	96.5	<0.0001	
Alcohol	Yes	10	4.55 (3.32–6.22)	92.4	<0.0001	0.59	8	2.05 (1.61–2.62)	68.1	0.003	0.83	9	2.96 (2.22–3.95)	92.1	<0.0001	0.36
	No	10	5.22 (3.80–7.17)	90.8	<0.0001		7	2.12 (1.57–2.85)	77.6	<0.0001		9	3.64 (2.47–5.37)	97.3	<0.0001	
Height	Yes	6	4.69 (2.95–7.44)	93.9	<0.0001	0.82	4	1.80 (1.36–2.38)	53.8	0.09	0.32	5	2.65 (1.75–4.03)	92.7	<0.0001	0.22
	No	14	4.97 (3.88–6.37)	90.4	<0.0001		11	2.22 (1.81–2.74)	70.8	<0.0001		13	3.56 (2.66–4.78)	96.5	<0.0001	
BMI, weight, adiposity	Yes	9	5.53 (3.81–8.03)	94.5	<0.0001	0.37	7	2.32 (1.74–3.09)	82.9	<0.0001	0.20	8	3.34 (2.38–4.68)	95.2	<0.0001	0.88
	No	11	4.33 (3.37–5.56)	86.6	<0.0001		8	1.89 (1.65–2.17)	0	0.89		10	3.25 (2.33–4.54)	95.8	<0.0001	
Physical activity	Yes	4	4.85 (1.89–12.48)	97.6	<0.0001	0.99	3	2.02 (0.79–5.20)	93.1	<0.0001	0.80	3	3.01 (0.93–9.77)	98.3	<0.0001	0.77
	No	16	4.79 (3.99–5.75)	84.9	<0.0001		12	2.12 (1.86–2.42)	37.1	0.09		15	3.33 (2.64–4.20)	95.1	<0.0001	
Cardiovascular disease	Yes	6	5.50 (4.19–7.24)	81.2	<0.0001	0.56	4	2.43 (2.00–2.96)	49.8	0.11	0.28	5	3.63 (2.86–4.60)	85.7	<0.0001	0.68
	No	14	4.66 (3.47–6.25)	93.3	<0.0001		11	1.92 (1.49–2.49)	74.2	<0.0001		13	3.21 (2.32–4.43)	96.5	<0.0001	
Hypertension	Yes	10	5.79 (4.32–7.77)	90.3	<0.0001	0.16	9	2.41 (1.97–2.95)	65.7	0.003	0.05	10	3.69 (2.96–4.59)	88.0	<0.0001	0.29
	No	10	4.09 (2.95–5.68)	92.5	<0.0001		6	1.71 (1.41–2.07)	26.7	0.23		8	2.94 (2.00–4.33)	96.9	<0.0001	
Hyperchole-sterolemia	Yes	7	5.83 (3.97–8.57)	92.4	<0.0001	0.28	6	2.53 (1.96–3.28)	73.1	0.002	0.04	7	3.61 (2.66–4.88)	91.2	<0.0001	0.51
	No	13	4.37 (3.36–5.69)	90.9	<0.0001		9	1.83 (1.61–2.09)	7.1	0.38		11	3.12 (2.29–4.24)	96.1	<0.0001	
Serum cholesterol	Yes	7	4.01 (2.74–5.87)	91.6	<0.0001	0.24	7	1.83 (1.54–2.18)	19.8	0.28	0.16	7	2.98 (2.16–4.11)	90.6	<0.0001	0.48
	No	13	5.44 (4.28–6.92)	89.3	<0.0001		8	2.34 (1.82–3.01)	77.0	<0.0001		11	3.49 (2.51–4.85)	97.0	<0.0001	
Diabetes	Yes	6	5.51 (3.49–8.69)	95.7	<0.0001	0.48	6	2.37 (1.76–3.18)	85.5	<0.0001	0.16	7	3.39 (2.38–4.83)	95.9	<0.0001	0.80
	No	14	4.49 (3.58–5.63)	85.2	<0.0001		9	1.88 (1.64–2.16)	0	0.93		11	3.22 (2.34–4.44)	95.3	<0.0001	

*n* denotes the number of studies.

^1^P for heterogeneity within each subgroup.

^2^P for heterogeneity between subgroups with meta-regression analysis.

^3^P for heterogeneity between men and women (excluding studies of men and women combined) with meta-regression analysis.

In sensitivity analyses where each study was excluded from the analysis at a time the association between current, former and ever versus never smoking and abdominal aortic aneurysms was robust (Supplementary Tables 4–6).

The mean (median) study quality scores were 6.6 (7.0) for the studies of current smokers, 6.8 (7.0) for former smokers, and 6.6 (7.0).

## Discussion

This meta-analysis of twenty two cohort studies confirm that smoking is a strong risk factor for abdominal aortic aneurysms, with 5-fold, 2-fold and 3.3-fold increases in the risk among current, former and ever smokers compared to never smokers, respectively. In addition, there was a strong dose-response relation between increasing number of cigarettes per day and pack-years and increasing risk of abdominal aortic aneurysms. The nonlinearity observed in the latter analyses may have been due to a modest number of studies included in the analyses and few data points at higher levels of cigarettes per day and pack-years of smoking. In addition, we found a linear inverse association between increasing duration of smoking cessation and risk of abdominal aortic aneurysms, with risk approaching that of never smokers at 25 years of smoking cessation.

Our study has some limitations which could have affected the results. Although publication bias can affect meta-analyses of published literature we found no evidence of publication bias in the current meta-analysis. Heterogeneity between studies was high in most of the analyses, however, because all the studies found risk estimates in the direction of increased risk the heterogeneity appeared to be driven more by differences in the sizes of the risk estimates rather than differences in the direction or presence or absence of an association. Smokers oftentimes have a less healthy lifestyle than non-smokers with lower physical activity, more abdominal adiposity, and unhealthier diets, however, we found that the associations persisted across subgroup analyses of adjustment for physical activity, adiposity and other established risk factors. Although the studies did not adjust for dietary factors, few dietary risk factors have been established and associations observed with dietary intake have generally been much weaker than those found in the current meta-analysis^48^. Although residual confounding by other risk factors or unidentified risk factors cannot entirely be excluded, the strong dose-response relation by smoking status (stronger association in current and ever smokers than former smokers), cigarettes per day and pack-years as well as the reduced risk with increasing duration of smoking cessation provides strong epidemiological support for a causal interpretation of the observed association between smoking and increased risk of abdominal aortic aneurysms.

In addition, a growing body of mechanistic studies support an association between smoking and the development of abdominal aortic aneurysms. Abdominal aortic aneurysms are characterized by loss of normal medial arterial structure and the near complete absence of normal lamellar elastin matrix^49^. The breakdown of the elastin and collagen of the arterial media is mediated by matrix metalloproteinases released by macrophages, and is also related to chronic inflammatory infiltration and loss or dysfunction of parenchymal cells central to matrix deposition and repair^49^. Smoking may activate tissue plasminogen activator which induces production of matrix metalloproteinases by macrophages and has also shown to disrupt collagen synthesis^50–52^. A rat study showed that nicotine administration weakened the vascular wall, increased gelatinolytic activity and promoted the destruction of elastin and collagen in the abdominal aorta and led to upregulation of matrix metalloproteinase-12 expression^53^, although another study found that smoking-induced aortic dilatation was not related to expression of matrix metalloproteinase-9 and -12^54^. Other experimental studies have found that both tobacco smoke and benzopyrene, a compound found in cigarette smoke, increased aortic muscular cell apoptosis and aortic macrophage infiltration and expression of matrix metalloproteinases-2, 9 and 12 and nuclear factor-κB and increased formation of abdominal aortic aneurysms in angiotensin-2 induced hypertension^55,56^.

Our study also has several strengths including the prospective design of the included studies (which avoids recall bias and reduces the potential for selection bias), the consistency of the findings across a range of subgroup and sensitivity analyses, and the moderately high study quality of the included studies. The large sample size (with >7800–8900 cases and 3–4.7 million participants) provided robust risk estimates of the relationship between tobacco smoking and abdominal aortic aneurysms. Given the ageing population, the strong associations observed between smoking and abdominal aortic aneurysms and the poor survival of patients with the disease the current findings provide strong support for interventions and policies to curb the tobacco epidemic.

## Conclusion

There was a 5-fold and 2-fold increase in the risk of abdominal aortic aneurysms among current and former smokers compared to never smokers, respectively. A positive dose-response relationship was observed between increasing number of cigarettes smoked per day and pack-years smoked and the risk of abdominal aortic aneurysm, while there was a reduced risk with increasing duration of smoking cessation with a risk similar to that of never smokers by 25 years of smoking cessation. This together with supportive experimental data provides strong evidence for a causal relationship between smoking and abdominal aortic aneurysms. The findings provide further support for interventions and policies to curb the global tobacco epidemic.

## Electronic supplementary material

Supplementary Material
