# Supplementary material for: Tobacco smoking and the risk of abdominal aortic aneurysm: a systematic review and meta-analysis of prospective studies
Source: Sci Rep. 2018 Oct 3;8:14786. doi: 10.1038/s41598-018-32100-2 (PMC6170425; doi:10.1038/s41598-018-32100-2)
Supplement: Supplementary file 1 — Supplementary Material [file 41598_2018_32100_MOESM1_ESM.pdf]

Supplement for: Dagfinn Aune, Sabrina Schlesinger, Teresa Norat, Elio Riboli. Tobacco smoking and the risk of abdominal aortic aneurysm - a systematic review and meta-analysis of prospective studies.

Supplementary text. Search terms used for PubMed and Embase search

PubMed:

(cigarette OR tobacco OR smoking OR smoke OR pipe OR cigar OR ETS betel nut OR bidi) AND ("aortic aneurysm")

Embase:

(((cigarette OR tobacco OR smoking OR smoke OR pipe OR cigar OR ETS betel nut OR bidi)ab.ti OR (cigarette/ OR tobacco/ OR smoking/ OR smoke/ OR pipe/ OR cigar/ OR ETS OR betel nut/ OR bidi)) AND ((aortic aneurysm)ab.ti OR aortic aneurysm/))

Supplementary Table 1. List of excluded studies and exclusion reason

Exclusion reason	Reference number
Abstract only publication	(1-7)
Case-control study	(8-27)
Case only study	(28-30)
Commentary, editorial, letter	(31-39)
Cross-sectional study	(40-83)
Duplicates	(84-88)
Meta-analysis	(89)
No confidence intervals	(90;91)
No risk estimates	(92-101)
Not relevant data	(102-107)
Not relevant exposure	(108-117)
Not relevant outcome	(118-121)
Patient populations	(122-126)
Review	(127-151)
Smoker population	(152;153)

Reference List

(1) Rodriguez S, I, Chasco J, Ibanez Y, Barrio I, Zugazabeitia G, Onaindia J, Laraudogoitia E, Lekuona I. Abdominal aortic aneurysm screening in a selected population of 600 patients referred for transthoracic echocardiography. European Heart Journal Conference: European Society of Cardiology, ESC Congress 2011;P534.

(2) Robson J, Kiran A, Maskell J, Hutchings A, Arden NK, Dasgupta B, Hamilton W, Emin A, Culliford D, Luqmani RA. The relative risk of aortic aneurysm in patients with giant cell arteritis compared with the general population. Arthritis and Rheumatism Conference: American College of Rheumatology/Association of Rheumatology Health Professionals Annual Scientific Meeting, ACR/ARHP 2013;Abstract number 895.

(3) Robson J, Kiran A, Maskell J, Arden N, Hutchings A, Emin A, Culliford D, Dasgupta B, Hamilton W, Luqmani R. Predictors of aortic aneurysms for giant cell arteritis subjects. Rheumatology (United Kingdom) Conference: British Society for Rheumatology and British Health Professionals in Rheumatology Annual Meeting 2013;Abstract.

(4) White W, McHugh SM, O'Halloran P, Murphy B, Boyle E, Allen M, Naughton P, Moneley D, Leahy A. Risk factors associated with the diagnosis of abdominal aortic aneurysm in an Irish screened population. Irish Journal of Medical Science Conference: 22nd Sylvester O'Halloran Meeting 2014;(var.pagings):February.

(5) Tang W, Alonso A, Lutsey PL, Lederle F, Yao L, Guan W, Folsom AR. Associations between middle-age risk factors and risk of asymptomatic abdominal aortic aneurysm: The atherosclerosis risk in communities (ARIC) study. Circulation Conference: American Heart Association's Epidemiology and Prevention/Nutrition, Physical Activity, and Metabolism 2014;129:AP341.

(6) Yao L, Alonso A, Lutsey P, Cheng S, Folsom A, Tang W. Associations between subclinical carotid atherosclerosis and future risk of abdominal aortic aneurysm: The atherosclerosis risk in community (ARIC) study. Circulation Conference: American Heart Association's Epidemiology and Prevention/Nutrition, Physical Activity, and Metabolism 2014;129(Suppl. 1):P340.

(7) Robson J, Kiran A, Maskell J, Hutchings A, Arden N, Dasgupta B, Hamilton W, Culliford D, Emin A, Luqmani R. The relative risk of aortic aneurysm in patients with giant cell arteritis compared with the general population of the UK. Presse Medicale Conference: 16th International Vasculitis and ANCA Workshop Paris France Conference Publication 2013;42(4 PART 2):672-3.

(8) Lamorte WW, Scott TE, Menzoian JO. Racial differences in the incidence of femoral bypass and abdominal aortic aneurysmectomy in Massachusetts: relationship to cardiovascular risk factors. J Vasc Surg 1995 Mar;21(3):422-31.

(9) Cole CW, Hill GB, Millar WJ, Laupacis A, Johnston KW. Selective screening for abdominal aortic aneurysm. Chronic Dis Can 1996;17(2):51-5.

(10) Blann AD, Devine C, Amiral J, McCollum CN. Soluble adhesion molecules, endothelial markers and atherosclerosis risk factors in abdominal aortic aneurysm: a comparison with claudicants and healthy controls. Blood Coagul Fibrinolysis 1998 Sep;9(6):479-84.

(11) Naydeck BL, Sutton-Tyrrell K, Schiller KD, Newman AB, Kuller LH. Prevalence and risk factors for abdominal aortic aneurysms in older adults with and without isolated systolic hypertension. Am J Cardiol 1999 Mar 1;83(5):759-64.

(12) Blanchard JF, Armenian HK, Friesen PP. Risk factors for abdominal aortic aneurysm: results of a case-control study. Am J Epidemiol 2000 Mar 15;151(6):575-83.

(13) Wanhainen A, Bergqvist D, Boman K, Nilsson TK, Rutegard J, Bjorck M. Risk factors associated with abdominal aortic aneurysm: a population-based study with historical and current data. J Vasc Surg 2005 Mar;41(3):390-6.

(14) Baumgartner I, Hirsch AT, Abola MTB, Cacoub PP, Poldermans D, Steg PG, Creager MA, Bhatt DL. Cardiovascular risk profile and outcome of patients with abdominal aortic aneurysm in out-patients with atherothrombosis: Data from the Reduction of Atherothrombosis for Continued Health (REACH) Registry. Journal of Vascular Surgery 2008;48(4):808-14.

(15) Badger SA, O'Donnell ME, Sharif MA, McMaster C, Young IS, Soong CV. The role of smoking in abdominal aortic aneurysm development. Angiology 2009 Feb;60(1):115-9.

(16) Meijer CA, Kokje VB, van Tongeren RB, Hamming JF, van Bockel JH, Moller GM, Lindeman JH. An association between chronic obstructive pulmonary disease and abdominal aortic aneurysm beyond smoking: results from a case-control study. Eur J Vasc Endovasc Surg 2012 Aug;44(2):153-7.

(17) Smelser DT, Tromp G, Elmore JR, Kuivaniemi H, Franklin DP, Kirchner HL, Carey DJ. Population risk factor estimates for abdominal aortic aneurysm from electronic medical records: a case control study. BMC Cardiovasc Disord 2014 Dec 4;14:174.

(18) Zuo SW, Wei YQ, Chen F, Chen DF, Wu T, Liu K, Sun KX, Juan J, Xiong J, Guo W. [A matched case-control study of risk factors in abdominal aortic aneurysm]. Beijing Da Xue Xue Bao 2014 Jun 18;46(3):412-6.

(19) Yuan H, Han X, Jiao D, Zhou P. A Case-Control Study of Risk Factors of Abdominal Aortic Aneurysm. Heart Surg Forum 2016 Oct 21;19(5):E224-E228.

(20) Liu J, Zuo SW, Li Y, Jia X, Jia SH, Zhang T, Song YX, Wei YQ, Xiong J, Hu YH, Guo W. Hyperhomocysteinaemia is an independent risk factor of abdominal aortic aneurysm in a Chinese Han population. Scientific reports 2016;6:17966.

(21) Zuo S, Xiong J, Wei Y, Chen D, Chen F, Liu K, Wu T, Hu Y, Guo W. Potential Interactions Between Genetic Polymorphisms of the Transforming Growth Factor-beta Pathway and Environmental Factors in Abdominal Aortic Aneurysms. European Journal of Vascular and Endovascular Surgery 50 (1) (pp 71-77), 2015;50(1):71-7.

(22) Powell JT, Worrell P, MacSweeney ST, Franks PJ, Greenhalgh RM. Smoking as a risk factor for abdominal aortic aneurysm. Ann N Y Acad Sci 1996 Nov 18;800:246-8.

(23) Madaric J, Vulev I, Bartunek J, Mistrik A, Verhamme K, De BB, Riecansky I. Frequency of abdominal aortic aneurysm in patients >60 years of age with coronary artery disease. Am J Cardiol 2005 Nov 1;96(9):1214-6.

(24) Ferro CR, de O, Guerra FF, de Lucena AJ, Nunes FP, Ortiz ST, Egito ES, de Sousa LC, Jatene AD, Piegas LS. Prevalence and risk factors for combined coronary artery disease and aortic aneurysm. Arq Bras Cardiol 2007 Jan;88(1):40-4.

(25) Miyazawa N, Akiyama I, Yamagata Z. Risk factors for the association of intracranial and aortic aneurysms. Acta Neurochir (Wien) 2007 Mar;149(3):221-9.

(26) Palazzuoli A, Gallotta M, Guerrieri G, Quatrini I, Franci B, Campagna MS, Neri E, Benvenuti A, Sassi C, Nuti R. Prevalence of risk factors, coronary and systemic atherosclerosis in abdominal aortic aneurysm: comparison with high cardiovascular risk population. Vasc Health Risk Manag 2008;4(4):877-83.

(27) Wilmink TB, Quick CR, Day NE. The association between cigarette smoking and abdominal aortic aneurysms. J Vasc Surg 1999 Dec;30(6):1099-105.

(28) MacSweeney ST, Ellis M, Worrell PC, Greenhalgh RM, Powell JT. Smoking and growth rate of small abdominal aortic aneurysms. Lancet 1994 Sep 3;344(8923):651-2.

(29) Rasmussen TE, Hallett JW, Jr., Tazelaar HD, Miller VM, Schulte S, O'Fallon WM, Weyand CM. Human leukocyte antigen class II immune response genes, female gender, and cigarette smoking as risk and modulating factors in abdominal aortic aneurysms. J Vasc Surg 2002 May;35(5):988-93.

(30) Gutierrez PS, Leite TN, Mangione FM. Male gender and smoking are related to single, but not to multiple, human aortic aneurysms. Cardiovasc Pathol 2015 Sep;24(5):290-3.

(31) Steinmann AF, Caramore W. Abdominal aortic aneurysm. The New England Journal of Medicine 1993;329(17):1276-7.

(32) Coggon D, Winter P, Martyn C, Inskip H. Contrasting epidemiology of aortic aneurysm and peripheral vascular disease in England and Wales. British Medical Journal 1996;312(7036):948.

(33) Takagi H, Umemoto T. How cigarette smoke accelerates abdominal aortic aneurysm. J Am Coll Surg 2005 Jul;201(1):149-50.

(34) Paraskevas KI, Stathopoulos V, Mikhailidis DP, Perrea D. Smoking, abdominal aortic aneurysms, and ischemic heart disease: is there a link? Angiology 2008 Dec 20;59(6):664-6.

(35) Powell JT, Norman PE. Abdominal aortic aneurysm events in postmenopausal women. BMJ 2008 Oct 14;337:a1894.

(36) Dua MM, Dalman RL. Identifying abdominal aortic aneurysm risk factors in postmenopausal women. Womens Health (Lond) 2009 Jan;5(1):33-7.

(37) Summaries for patients. Screening for abdominal aortic aneurysm: U.S. Preventive Services Task Force recommendation statement. Annals of internal medicine 2014;161(4):I-26.

(38) Schooling CM. Smoking, sex, risk factors and abdominal aortic aneurysm: is it all down to testosterone? J Epidemiol Community Health 2015 May;69(5):495.

(39) Harrison SC, Humphries SE. Regarding "analysis of risk factors for abdominal aortic aneurysm in a cohort of more than 3 million individuals". J Vasc Surg 2011 Jan;53(1):263-4.

(40) Horiuchi Y, Tanimoto S, Aoki J, Asami M, Nakajima H, Tooda E, Hara K, Tanabe K. Abdominal aortic aneurysm in patients with acute myocardial infarction: Prevalence and risk factors. International Journal of Cardiology 2016;205:56-7.

(41) Singh K, Bonaa KH, Jacobsen BK, Bjork L, Solberg S. Prevalence of and risk factors for abdominal aortic aneurysms in a population-based study: The Tromso study. American Journal of Epidemiology 2001;154(3):236-44.

(42) Benoit AG, Campbell BI, Tanner JR, Staley JD, Wallbridge HR, Biehl DR, Bradley BD, Louridas G, Guzman RP, Fromm RA. Risk factors and prevalence of perioperative cognitive dysfunction in abdominal aneurysm patients. Journal of Vascular Surgery 2005;42(5):884-90.

(43) Badger SA, O'Donnell ME, Sharif MA, Boyd CS, Hannon RJ, Lau LL, Lee B, Soong CV. Risk factors for abdominal aortic aneurysm and the influence of social deprivation. Angiology 2008 Oct;59(5):559-66.

(44) Leong BDK, Zainal AA, Chuah JA, Voo SY. Prevalence of peripheral arterial disease and abdominal aortic aneurysm among patients with acute coronary syndrome. Medical Journal of Malaysia 2013;68(1):10-2.

(45) Durieux R, Van DH, Labropoulos N, Yazici A, Legrand V, Albert A, Defraigne J-O, Sakalihasan N. High Prevalence of abdominal aortic aneurysm in patients with three-vessel coronary artery disease. European Journal of Vascular and Endovascular Surgery 47 (3) (pp 273-278), 2014;47(3):273-8.

(46) Corrado G, Durante A, Genchi V, Trabattoni L, Beretta S, Rovelli E, Foglia-Manzillo G, Ferrari G. Prevalence of previously undiagnosed abdominal aortic aneurysms in the area of Como: the ComoCuore "looking for AAA" ultrasonography screening. International Journal of Cardiovascular Imaging 2016;32(8):1213-7.

(47) Killeen S, Neary P, O'Sullivan M, Redmond HP, Fulton G. Daily diurnal variation in admissions for ruptured abdominal aortic aneurysms. World journal of surgery 2007;31(9):1869-71.

(48) Waterhouse DF, Cahill RA. Simple adaptation of current abdominal aortic aneurysm screening programs may address all-cause cardiovascular mortality. Prospective observational cohort study. American Heart Journal 2008;155(5):938-45.

(49) Persson S-E, Boman K, Wanhainen A, Carlberg B, Arnerlov C. Decreasing prevalence of abdominal aortic aneurysm and changes in cardiovascular risk factors. Journal of Vascular Surgery 2017;65(3):651-8.

(50) Cho I-J, Jang S-Y, Chang H-J, Shin S, Shim CY, Hong G-R, Chung N. Aortic aneurysm screening in a high-risk population: A non-contrast computed tomography study in Korean males with hypertension. Korean Circulation Journal 2014;44(3):162-9.

(51) Thurmond AS, Semler HJ. Abdominal aortic aneurysm: Incidence in a population at risk. Journal of Cardiovascular Surgery 1986;27(4):457-60.

(52) Collin J, Araujo L, Walton J, Lindsell D. Oxford screening programme for abdominal aortic aneurysm in men aged 65 to 74 years. Lancet 1988 Sep 10;2(8611):613-5.

(53) O'Kelly TJ, Heather BP. General practice-based population screening for abdominal aortic aneurysms: a pilot study. Br J Surg 1989 May;76(5):479-80.

(54) Bengtsson H, Bergqvist D, Ekberg O, Janzon L. A population based screening of abdominal aortic aneurysms (AAA). Eur J Vasc Surg 1991 Feb;5(1):53-7.

(55) Adamson J, Powell JT, Greenhalgh RM. Selection for screening for familial aortic aneurysms. Br J Surg 1992 Sep;79(9):897-8.

(56) Fowl RJ, Blebea J, Stallion A, Marsch JT, Marsch JG, Love M, Patterson RB, Kempczinski RF. Prevalence of unsuspected abdominal aortic aneurysms in male veterans. Ann Vasc Surg 1993 Mar;7(2):117-21.

(57) Simoni G, Pastorino C, Perrone R, Ardia A, Gianrossi R, De CF, Cittadini G, Jr., Baiardi A, Bachi V. Screening for abdominal aortic aneurysms and associated risk factors in a general population. Eur J Vasc Endovasc Surg 1995 Aug;10(2):207-10.

(58) Roland CF, Radnich JJ. Screening a high-risk population for abdominal aortic aneurysm. J Vasc Nurs 1996 Jun;14(2):45-7.

(59) Lederle FA, Johnson GR, Wilson SE, Chute EP, Littooy FN, Bandyk D, Krupski WC, Barone GW, Acher CW, Ballard DJ. Prevalence and associations of abdominal aortic aneurysm detected through screening. Aneurysm Detection and Management (ADAM) Veterans Affairs Cooperative Study Group. Ann Intern Med 1997 Mar 15;126(6):441-9.

(60) Vazquez C, Sakalihasan N, D'Harcour JB, Limet R. Routine ultrasound screening for abdominal aortic aneurysm among 65- and 75-year-old men in a city of 200,000 inhabitants. Ann Vasc Surg 1998 Nov;12(6):544-9.

(61) Jamrozik K, Norman PE, Spencer CA, Parsons RW, Tuohy R, Lawrence-Brown MM, Dickinson JA. Screening for abdominal aortic aneurysm: lessons from a population-based study. Med J Aust 2000 Oct 2;173(7):345-50.

(62) Adachi K, Iwasawa T, Ono T. Screening for abdominal aortic aneurysms during a basic medical checkup in residents of a Japanese rural community. Surg Today 2000;30(7):594-9.

(63) Vardulaki KA, Walker NM, Day NE, Duffy SW, Ashton HA, Scott RA. Quantifying the risks of hypertension, age, sex and smoking in patients with abdominal aortic aneurysm. Br J Surg 2000 Feb;87(2):195-200.

(64) Lederle FA, Johnson GR, Wilson SE. Abdominal aortic aneurysm in women. J Vasc Surg 2001 Jul;34(1):122-6.

(65) Barba A, Estallo L, Rodriguez L, Baquer M, Vega de CM. Detection of abdominal aortic aneurysm in patients with peripheral artery disease. Eur J Vasc Endovasc Surg 2005 Nov;30(5):504-8.

(66) Derubertis BG, Trocciola SM, Ryer EJ, Pieracci FM, McKinsey JF, Faries PL, Kent KC. Abdominal aortic aneurysm in women: prevalence, risk factors, and implications for screening. J Vasc Surg 2007 Oct;46(4):630-5.

(67) Greco G, Egorova NN, Gelijns AC, Moskowitz AJ, Manganaro AJ, Zwolak RM, Riles TS, Kent KC. Development of a novel scoring tool for the identification of large >/=5 cm abdominal aortic aneurysms. Ann Surg 2010 Oct;252(4):675-82.

(68) Kent KC, Zwolak RM, Egorova NN, Riles TS, Manganaro A, Moskowitz AJ, Gelijns AC, Greco G. Analysis of risk factors for abdominal aortic aneurysm in a cohort of more than 3 million individuals. J Vasc Surg 2010 Sep;52(3):539-48.

(69) Svensjo S, Bjorck M, Gurtelschmid M, Djavani GK, Hellberg A, Wanhainen A. Low prevalence of abdominal aortic aneurysm among 65-year-old Swedish men indicates a change in the epidemiology of the disease. Circulation 2011 Sep 6;124(10):1118-23.

(70) Jawien A, Formankiewicz B, Derezinski T, Brazis P, Migdalski A, Piotrowicz R, Woda L, Gorecki DM. Preliminary results from the first Polish screening program for abdominal aortic aneurysm in the Kuyavian-Pomeranian Province. [Polish, English]. Acta Angiologica 2012;18(1):9-17.

(71) Svensjo S, Bjorck M, Wanhainen A. Current prevalence of abdominal aortic aneurysm in 70-year-old women. Br J Surg 2013 Feb;100(3):367-72.

(72) Chun KC, Teng KY, Chavez LA, Van Spyk EN, Samadzadeh KM, Carson JG, Lee ES. Risk factors associated with the diagnosis of abdominal aortic aneurysm in patients screened at a regional Veterans Affairs health care system. Ann Vasc Surg 2014 Jan;28(1):87-92.

(73) Jones GT, Hill BG, Curtis N, Kabir TD, Wong LE, Tilyard MW, Williams MJ, van Rij AM. Comparison of three targeted approaches to screening for abdominal aortic aneurysm based on cardiovascular risk. Br J Surg 2016 Aug;103(9):1139-46.

(74) Makrygiannis G, Labalue P, Erpicum M, Schlitz M, Seidel L, El HM, Gangolf M, Albert A, Defraigne JO, Lindholt JS, Sakalihasan N. Extending Abdominal Aortic Aneurysm Detection to Older Age Groups: Preliminary Results from the Liege Screening Programme. Ann Vasc Surg 2016 Oct;36:55-63.

(75) Chabok M, Nicolaides A, Aslam M, Farahmandfar M, Humphries K, Kermani NZ, Coltart J, Standfield N. Risk factors associated with increased prevalence of abdominal aortic aneurysm in women. Br J Surg 2016 Aug;103(9):1132-8.

(76) Salvador-Gonzalez B, Martin-Baranera M, Borque-Ortega A, Saez-Saez RM, de Albert-Delas VM, Carreno-Garcia E, Tarin-Masriera L, Badia-Millan P, Martinez-Gil M, Torrabadella-Fabrega J. Prevalence of Abdominal Aortic Aneurysm in Men Aged 65-74 Years in a Metropolitan Area in North-East Spain. Eur J Vasc Endovasc Surg 2016 Jul;52(1):75-81.

(77) Lindberg S, Zarrouk M, Holst J, Gottsater A. Inflammatory markers associated with abdominal aortic aneurysm. European Cytokine Network 2016;27(3):75-80.

(78) Li W, Luo S, Luo J, Liu Y, Ning B, Huang W, Xue L, Chen J. Predictors Associated With Increased Prevalence of Abdominal Aortic Aneurysm in Chinese Patients with Atherosclerotic Risk Factors. Eur J Vasc Endovasc Surg 2017 May 17;54(1):43-9.

(79) Derezinski TL, Formankiewicz B, Migdalski A, Brazis P, Jakubowski G, Woda L, Jawien A. Prevalence of abdominal aortic aneurysms in rural/urban population in central Poland. Kardiol Pol 2017 Apr 10;75(7):705-10.

(80) Han SA, Joh JH, Park HC. Risk Factors for Abdominal Aortic Aneurysm in the Korean Population. Ann Vasc Surg 2017 May;41:135-40.

(81) Brown LC, Powell JT. Risk factors for aneurysm rupture in patients kept under ultrasound surveillance. UK Small Aneurysm Trial Participants. Ann Surg 1999 Sep;230(3):289-96.

(82) Brosnan M, Collins CG, Moneley DS, Kelly CJ, Leahy AL. Making the case for cardiovascular screening in Irish males: detection of abdominal aortic aneurysms, and assessment of cardiovascular risk factors. Eur J Vasc Endovasc Surg 2009 Mar;37(3):300-4.

(83) Lopez-De-Andres A, Jimenez-Trujillo I, Jimenez-Garcia R, Hernandez-Barrera V, de Miguel-Yanes JM, Mendez-Bailon M, Perez-Farinos N, Salinero-Fort MA, Carrasco-Garrido P. National trends in incidence and outcomes of abdominal aortic aneurysm among elderly type 2 diabetic and non-diabetic patients in Spain (2003-2012). Cardiovascular Diabetology 2015;15(1):103.

(84) Hammond EC. Smoking in relation to mortality and morbidity. Findings in first thirty-four months of follow-up in a prospective study started in 1959. Journal of the National Cancer Institute 32 (5) (pp 1161-1188), 1964;1964.

(85) Robson JC, Kiran A, Maskell J, Hutchings A, Arden N, Dasgupta B, Hamilton W, Emin A, Culliford D, Luqmani RA. The relative risk of aortic aneurysm in patients with giant cell arteritis compared with the general population of the UK. Ann Rheum Dis 2015 Jan;74(1):129-35.

(86) Carstensen JM, Pershagen G, Eklund G. Mortality in relation to cigarette and pipe smoking: 16 years' observation of 25,000 Swedish men. J Epidemiol Community Health 1987 Jun;41(2):166-72.

(87) Stackelberg O, Wolk A, Eliasson K, Hellberg A, Bersztel A, Larsson SC, Orsini N, Wanhainen A, Bjorck M. Lifestyle and Risk of Screening-Detected Abdominal Aortic Aneurysm in Men. J Am Heart Assoc 2017 May 10;6(5):e004725.

(88) Hammond EC, Horn D. Smoking and death rates. Report on forty-four months of follow-up of 187,783 men. I. Total mortality. II. Death rates by cause. JAMA (Chicago, Ill) 1958;166(10):1159-72.

(89) Cornuz J, Sidoti PC, Tevaearai H, Egger M. Risk factors for asymptomatic abdominal aortic aneurysm: systematic review and meta-analysis of population-based screening studies. Eur J Public Health 2004 Dec;14(4):343-9.

(90) Weir JM, Dunn JE, Jr. Smoking and mortality: a prospective study. Cancer 1970 Jan;25(1):105-12.

(91) Rogot E, Murray JL. Smoking and causes of death among U.S. veterans: 16 years of observation. Public Health Rep 1980 May;95(3):213-22.

(92) Garfinkel L. The association between cigarette smoking and coronary heart disease and other vascular diseases. Bull N Acad Med 1968;44(12):1495-501.

(93) Auerbach O, Garfinkel L. Atherosclerosis and aneurysm of aorta in relation to smoking habits and age. Chest 1980;78(6):805-9.

(94) Alcorn HG, Wolfson SK, Jr., Sutton-Tyrrell K, Kuller LH, O'Leary D. Risk factors for abdominal aortic aneurysms in older adults enrolled in The Cardiovascular Health Study. Arterioscler Thromb Vasc Biol 1996 Aug;16(8):963-70.

(95) Brivady A, Normand B, Fabry R, Pochon P, Cheynel J. Prospective study by ultrasonography of abdominal aortic diameter in atherosclerosis of the lower limbs - The role of atheromatous disease - Report of 1,413 cases. Vascular Surgery 1997;31(1):51-65.

(96) Lindholt JS, Heickendorff L, Antonsen S, Fasting H, Henneberg EW. Natural history of abdominal aortic aneurysm with and without coexisting chronic obstructive pulmonary disease. Journal of Vascular Surgery 1998;28(2):226-33.

(97) Cho I-J, Heo R, Chang H-J, Shin S, Shim CY, Hong G-R, Min JK, Chung N. Correlation between coronary artery calcium score and aortic diameter in a high-risk population of elderly male hypertensive patients. Coronary Artery Disease 2014;25(8):698-704.

(98) Lederle FA, Noorbaloochi S, Nugent S, Taylor BC, Grill JP, Kohler TR, Cole L. Multicentre study of abdominal aortic aneurysm measurement and enlargement. The British journal of surgery 2015;102(12):1480-7.

(99) Jimenez-Trujillo I, Gonzalez-Pascual M, Jimenez-Garca R, Hernandez-Barrera V, Miguel-Yanes JM, Mendez-Bailon M, De Miguel-DIez J, ngel Salinero-Fort M, Perez-Farinos N, Carrasco-Garrido P, Lopez-De-Andres A. Type 2 diabetes mellitus and thoracic aortic aneurysm and dissection an observational population-based study in Spain from 2001 to 2012. Medicine (United States) 2016;95(18):e3618.

(100) Karakus A, Tasin V, Kartal I, Kuvandik G. Screening for abdominal aortic aneurysm in geriatric population. Journal of Clinical and Analytical Medicine 2017;8:114-6.

(101) Benson RA, Poole R, Murray S, Moxey P, Loftus IM. Screening results from a large United Kingdom abdominal aortic aneurysm screening center in the context of optimizing United Kingdom National Abdominal Aortic Aneurysm Screening Programme protocols. Journal of Vascular Surgery 63 (2 Supplement) (pp 301-304), 2016;63((2 Supplement)):301-4.

(102) Emdin CA, Anderson SG, Callender T, Conrad N, Salimi-Khorshidi G, Mohseni H, Woodward M, Rahimi K. Usual blood pressure, peripheral arterial disease, and vascular risk: Cohort study of 4.2 million adults. BMJ 2015;351:h4865.

(103) Nomura E, Suzuki A, Inoue I, Nakagawara J, Takahashi K, Takahashi T, Manabe Y, Yokota C, Okada K, Nishihara T, Yamamoto Y, Noda K, et al. Subsequent vascular events after ischemic stroke: The Japan statin treatment against recurrent stroke - Longitudinal. Journal of Stroke and Cerebrovascular Diseases 2015;24(2):473-9.

(104) Rodella LF, Rezzani R, Bonomini F, Peroni M, Cocchi MA, Hirtler L, Bonardelli S. Abdominal aortic aneurysm and histological, clinical, radiological correlation. Acta Histochemica 2016;118(3):256-62.

(105) Jamrozik K, Spencer CA, Lawrence-Brown MM, Norman PE. Does the Mediterranean paradox extend to abdominal aortic aneurysm? Int J Epidemiol 2001 Oct;30(5):1071-5.

(106) Garrafa E, Marengoni A, Nave RD, Caimi L, Cervi E, Giulini SM, Imberti L, Bonardelli S. Association between human parainfluenza virus type 1 and smoking history in patients with an abdominal aortic aneurysm. J Med Virol 2013 Jan;85(1):99-104.

(107) Olchanski N, Winn A, Cohen JT, Neumann PJ. Abdominal aortic aneurysm screening: How many life years lost from underuse of the medicare screening benefit? Journal of General Internal Medicine 2014;29(8):1155-61.

(108) Savji N, Rockman CB, Skolnick AH, Guo Y, Adelman MA, Riles T, Berger JS. Association between advanced age and vascular disease in different arterial territories: A population database of over 3.6 million subjects. Journal of the American College of Cardiology 2013;61(16):1736-43.

(109) Chun KC, Teng KY, Van Spyk EN, Carson JG, Lee ES. Outcomes of an abdominal aortic aneurysm screening program. Journal of Vascular Surgery 2013;57(2):376-81.

(110) George J, Rapsomaniki E, Pujades-Rodriguez M, Shah AD, Denaxas S, Herrett E, Smeeth L, Timmis A, Hemingway H. How does cardiovascular disease first present in women and men? Circulation 2015;132(14):1320-8.

(111) Hansen ML, Thomsen MD, Rasmussen LM, Lindholt JS. Abdominal aortic aneurysm, arterial stiffening and the role of the intraluminal thrombus. Vasa - European Journal of Vascular Medicine 2015;44(5):349-53.

(112) Chiu H-Y, Lo P-C, Huang W-F, Tsai Y-W, Tsai T-F. Increased risk of aortic aneurysm (AA) in relation to the severity of psoriasis: A national population-based matched-cohort study. Journal of the American Academy of Dermatology 2016;75(4):747-54.

(113) Ungprasert P, Crowson CS, Matteson EL. Risk of cardiovascular disease among patients with sarcoidosis: A population-based retrospective cohort study. Arthritis and Rheumatology Conference: American College of Rheumatology/Association of Rheumatology Health Professionals Annual Scientific Meeting, ACR/ARHP 2016;68 (Suppl. 10):-331.

(114) Khalid U, Egeberg A, Ahlehoff O, Smedegaard L, Gislason GH, Hansen PR. Nationwide study on the risk of abdominal aortic aneurysms in patients with psoriasis. Arteriosclerosis, Thrombosis, and Vascular Biology 2016;36(5):1043-8.

(115) Folsom AR, Yao L, Alonso A. Circulating biomarkers and abdominal aortic aneurysm incidence: The Atherosclerosis Risk in Communities (ARIC) study. Journal of Vascular Surgery 2016;63(3):846.

(116) Fagerberg B, Borne Y, Sallsten G, Smith JG, Acosta S, Persson M, Melander O, Forsgard N, Gottsater A, Hedblad B, Barregard L, Engstrom G. Circulating cadmium concentration and risk of aortic aneurysms: A nested case-control study within the Malmo Diet and Cancer cohort. Atherosclerosis 2017;261:37-43.

(117) Joshy G, Korda RJ, Attia J, Liu B, Bauman AE, Banks E. Body mass index and incident hospitalisation for cardiovascular disease in 158 546 participants from the 45 and Up Study. International Journal of Obesity 2014;38(6):848-56.

(118) Takeuchi T, Adachi H, Ohuchida M, Nakamura T, Satoh A, Jacobs DR, Jr., Imaizumi T. A case-control study found that low albumin and smoking were associated with aortic dissection. J Clin Epidemiol 2004 Apr;57(4):386-91.

(119) Lottman PEM, van Marrewijk CJ, Fransen GAJ, Laheij RJF, Buth J. Impact of smoking of endovascular abdominal aortic aneurysm surgery outcome. European Journal of Vascular and Endovascular Surgery 2004;27(5):512-8.

(120) Gokani VJ, Sidloff D, Bath MF, Bown MJ, Sayers RD, Choke E. A retrospective study: Factors associated with the risk of abdominal aortic aneurysm rupture. Vascular Pharmacology 2015;65:13-6.

(121) Bhak RH, Wininger M, Johnson GR, Lederle FA, Messina LM, Ballard DJ, Wilson SE. Factors associated with small abdominal aortic aneurysm expansion rate. JAMA Surgery 2015;150(1):44-50.

(122) Yii MK. Epidemiology of abdominal aortic aneurysm in an Asian population. ANZ J Surg 2003 Jun;73(6):393-5.

(123) Brady AR, Thompson SG, Fowkes FG, Greenhalgh RM, Powell JT. Abdominal aortic aneurysm expansion: risk factors and time intervals for surveillance. Circulation 2004 Jul 6;110(1):16-21.

(124) Weijmans M, van der GY, de Borst GJ, Asselbergs FW, Cramer MJ, Algra A, Visseren FL. The relation between the presence of cardiovascular disease and vascular risk factors in offspring and the occurrence of new vascular events in their parents already at high vascular risk. Am Heart J 2015 Oct;170(4):744-52.

(125) Mearini L, Zucchi A, Pizzirusso G, Vivacqua C, Mearini E. Incidence and evolution of aortic aneurysm in patients with bladder cancer. Archivio Italiano di Urologia e Andrologia 2004;76(2):80-2.

(126) Vanni V, Hernesniemi J, Turtiainen M, Turtiainen J, Hakala T. Screening Men with Coronary Heart Disease for Abdominal Aortic Aneurysm: A Prospective Cohort Study. World journal of surgery 2015;39(9):2354-8.

(127) Flessenkamper I, Sollner H, Eckstein H-H. Clinical risk indicators for formation of abdominal aortic aneurysms: English version. Gefasschirurgie 1920;(1):2015.

(128) McGill HC, Jr. The cardiovascular pathology of smoking. Am Heart J 1988 Jan;115(1 Pt 2):250-7.

(129) Reilly JM, Tilson MD. Incidence and etiology of abdominal aortic aneurysms. Surg Clin North Am 1989 Aug;69(4):705-11.

(130) MacSweeney ST, Powell JT, Greenhalgh RM. Pathogenesis of abdominal aortic aneurysm. Br J Surg 1994 Jul;81(7):935-41.

(131) Wald NJ, Hackshaw AK. Cigarette smoking: an epidemiological overview. Br Med Bull 1996 Jan;52(1):3-11.

(132) Taylor BV, Oudit GY, Kalman PG, Liu P. Clinical and pathophysiological effects of active and passive smoking on the cardiovascular system. Can J Cardiol 1998 Sep;14(9):1129-39.

(133) Wilmink AB, Quick CR. Epidemiology and potential for prevention of abdominal aortic aneurysm. Br J Surg 1998 Feb;85(2):155-62.

(134) Blanchard JF. Epidemiology of abdominal aortic aneurysms. Epidemiol Rev 1999;21(2):207-21.

(135) Burns DM. Epidemiology of smoking-induced cardiovascular disease. Prog Cardiovasc Dis 2003 Jul;46(1):11-29.

(136) Lederle FA, Nelson DB, Joseph AM. Smokers' relative risk for aortic aneurysm compared with other smoking-related diseases: a systematic review. J Vasc Surg 2003 Aug;38(2):329-34.

(137) Crawford CM, Hurtgen-Grace K, Talarico E, Marley J. Abdominal aortic aneurysm: an illustrated narrative review. J Manipulative Physiol Ther 2003 Mar;26(3):184-95.

(138) Prisant LM, Mondy JS, III. Abdominal aortic aneurysm. J Clin Hypertens (Greenwich) 2004 Feb;6(2):85-9.

(139) Rempher KJ. Cardiovascular sequelae of tobacco smoking. Crit Care Nurs Clin North Am 2006 Mar;18(1):13-20.

(140) Kakafika AI, Mikhailidis DP. Smoking and aortic diseases. Circ J 2007 Aug;71(8):1173-80.

(141) Curci JA. Effect of smoking on abdominal aortic aneurysms: novel insights through murine models. Future Cardiol 2007 Jul;3(4):457-66.

(142) Bullen C. Impact of tobacco smoking and smoking cessation on cardiovascular risk and disease. Expert Rev Cardiovasc Ther 2008 Jul;6(6):883-95.

(143) Hannawa KK, Eliason JL, Upchurch GR, Jr. Gender differences in abdominal aortic aneurysms. Vascular 2009 May;17 Suppl 1:S30-S39.

(144) Fernandez-Moure JS, Vykoukal D, Davies MG. Biology of aortic aneurysms and dissections. Methodist Debakey Cardiovasc J 2011 Jul;7(3):2-7.

(145) Aggarwal S, Qamar A, Sharma V, Sharma A. Abdominal aortic aneurysm: A comprehensive review. Exp Clin Cardiol 2011;16(1):11-5.

(146) Sugamura K, Keaney JF, Jr. Nicotine: linking smoking to abdominal aneurysms. Nat Med 2012 Jun 6;18(6):856-8.

(147) Kuivaniemi H, Elmore JR. Opportunities in abdominal aortic aneurysm research: Epidemiology, genetics, and pathophysiology. Annals of Vascular Surgery 2012;26(6):862-70.

(148) Katsiki N, Papadopoulou SK, Fachantidou AI, Mikhailidis DP. Smoking and vascular risk: are all forms of smoking harmful to all types of vascular disease? Public Health 2013 May;127(5):435-41.

(149) Keisler B, Carter C. Abdominal aortic aneurysm. American Family Physician 2015;91(8):538-43.

(150) Soderberg P, Wanhainen A, Svensjo S. Five Year Natural History of Screening Detected Sub-Aneurysms and Abdominal Aortic Aneurysms in 70 Year Old Women and Systematic Review of Repair Rate in Women. Eur J Vasc Endovasc Surg 2017 Jun;53(6):802-9.

(151) Reilly MP. Tobacco-related cardiovascular diseases in the 21st century. Arterioscler Thromb Vasc Biol 2013 Jul;33(7):1458-9.

(152) Tornwall ME, Virtamo J, Haukka JK, Albanes D, Huttunen JK. Life-style factors and risk for abdominal aortic aneurysm in a cohort of Finnish male smokers. Epidemiology 2001 Jan;12(1):94-100.

(153) Franks PJ, Edwards RJ, Greenhalgh RM, Powell JT. Risk factors for abdominal aortic aneurysms in smokers. Eur J Vasc Endovasc Surg 1996 May;11(4):487-92.

Supplementary Table 2. Prospective studies of smoking and abdominal aortic aneurysm

First author, publication year, country	Study name or description	Study period	Number of participants, number of cases	Type of Smoking, subgroup	Comparison	Relative risk (95% confidence interval)	Adjustment for confounders
Hammond EC et al, 1966, USA	Cancer Prevention Study 1	1959-1960 - 1962, 3.75 years	257198 men, age 45-79 years and 362398 women, age 45-64 years: 314/23 AAA deaths	Smoking status, age 45-64 years, men<br>Smoking status, age 65-79 years, Men<br>Smoking status, age 45-64 years, Women	Never<br>Ever<br>Never<br>Ever<br>Never<br>Ever	1.00<br>2.62 (1.60-4.28)<br>1.00<br>4.92 (3.01-8.04)<br>1.00<br>3.89 (1.72-8.82)	Age
Hammond EC et al, 1969, USA	Cancer Prevention Study 1	1959-1960 - NA, 6 years follow-up	218435 men, age 50-69 years: 260 AAA deaths	Smoking status and cigarettes per day	Never<br>Current, 1-9 cig/d<br>Current, 10-19<br>Current, 20-39<br>Current, 40+	1.00<br>2.62 (1.38-4.96)<br>3.85 (2.09-7.09)<br>4.54 (2.49-8.28)<br>8.00 (4.50-14.24)	Age
Strachan DP et al, 1991, United Kingdom	The Whitehall Study	1967-1969 - 1987, 16.6 years follow-up	18403 men, age 40-64 years: 99 AA cases	Smoking status, all AA	Never<br>Former<br>Current, pipe/cigar<br>Current, manufactured cig.<br>Current, hand-rolled	1.0<br>1.3 (0.42-4.1)<br>6.7 (1.7-26.5)<br>6.5 (2.3-18.7)<br>25.0 (7.5-83.3)	DBP
				Pipe/cigar smoking, all AA	Never<br>Current	1.0<br>5.4 (1.9-15.3)	
				Pipe/cigar smoking, dissecting AA	Never<br>Current	1.0<br>16.7 (3.4-82.1)	
				Pipe/cigar smoking, AAA	Never<br>Current	1.0<br>2.4 (0.3-22.5)	
				Pipe/cigar smoking, other AA	Never<br>Current	1.0<br>No cases	
				Cigarette smoking (manufactured), all AA	Never<br>Current	1.0<br>5.3 (3.1-9.1)	
				Cigarette smoking (manufactured), dissecting AA	Never<br>Current	1.0<br>7.7 (2.8-21.7)	
				Cigarette smoking (manufactured), AAA	Never<br>Current	1.0<br>4.6 (2.1-10.3)	
				Cigarette smoking (manufactured), other AA	Never<br>Current	1.0<br>5.3 (1.7-17.3)	

				Cigarette smoking (hand-rolled), all AA<br>Cigarette smoking (hand-rolled), dissecting AA<br>Cigarette smoking (hand-rolled), AAA<br>Cigarette smoking (hand-rolled), other AA	Never<br>Current<br>Never<br>Current<br>Never<br>Current<br>Never<br>Current	1.0<br>20.1 (9.2-43.8)<br>1.0<br>56.5 (13.0-246.0)<br>1.0<br>14.6 (4.1-51.7)<br>1.0<br>33.6 (5.4-210.0)	
Goldberg RJ et al, 1995, USA	Honolulu Heart Program	1965-1988, 23 years follow-up	2710 men, age 55-64 years: 119 AA cases	Cigarettes per day	Nonsmoker<br><20 cig/d<br>20<br>>20	1.00<br>2.54 (1.00-6.45)<br>3.38 (1.68-6.78)<br>3.56 (1.70-7.45)	Ventricular rate, BMI, SBP, serum cholesterol, TG, serum glucose, serum uric acid, hematocrit, forced expiratory volume, physical activity, alcohol
Lee AJ et al, 1997, United Kingdom	Edinburgh Artery Study	NA-1992-1994, 5 years follow-up	1592 men and women, age 55-74 years: 40 AAA cases 200 controls (nested case-control study)	Smoking status	Never/quit >5 years<br>Current/former ≤5 years	1.00<br>3.08 (1.53-6.21)	Age, sex
Nilsson S et al, 2001, Sweden	Swedish 1963 Smoking Habit Survey	1963 – 1996, 33 years follow-up	27841 men and 28089 women, age 18-69 years: 196 AAA deaths	Smoking status, men<br><br><br>Cigarettes per day<br><br><br><br><br>Smoking status, women<br><br><br>Cigarettes per day	Never<br>Former<br>Current<br>Never<br>1-7 cig/d<br>8-15<br>16-25<br>>25<br>Never<br>Former<br>Current<br>Never<br>1-7 cig/d<br>8-15	1.00<br>1.57 (0.94-2.63)<br>3.30 (2.08-5.23)<br>1.00<br>2.58 (1.41-4.71)<br>4.20 (2.36-7.46)<br>3.76 (1.80-7.86)<br>2.15 (0.29-16.0)<br>1.00<br>0.42 (0.06-3.02)<br>3.43 (2.11-5.59)<br>1.00<br>2.54 (1.34-4.80)<br>6.14 (3.35-11.3)	Age, residence
Rodin MB et al, 2003, USA	Chicago Heart Association Detection	1967-1973 - 2000, 30 years	10574 men and 8700 women, age 40-64 years:	Smoking status	Never<br>Former<br>Current	1.00<br>2.01 (1.42-2.85)<br>5.18 (3.85-6.96)	Age, sex, height, DBP, serum cholesterol

	Project in Industry Cohort	follow-up	309/109 AAA cases				
Lindblad B et al, 2005, Sweden	Malmo Preventive Project	1974-1991, 21 years follow-up	22444 men and 10982 women, mean age 43.7 years: 126/6 AAA cases	Smoking	Never/former<br>Current	1.00<br>3.51 (1.92-6.44)	TG, DBP, serum cholesterol, physical inactivity
Iribarren C et al, 2007, USA	Kaiser Permanente Mulitphasic Health Checkups	1965-1970 - 2003, 13 years follow-up	104813 men and women, age ≥18 years: 605 AAA cases	Cigarette smoking status	Never<br>Former<br>Current, <1 pack/d<br>Current, 1-2 packs/d<br>Current, ≥3 packs/d<br>Unknown packs/d<br>Unknown	1.00<br>2.07 (1.59-2.69)<br>2.79 (2.09-3.73)<br>5.11 (3.96-6.60)<br>5.17 (3.72-7.18)<br>3.13 (0.99-9.87)<br>3.68 (1.33-10.17)	Age, sex, race, education, alcohol, height, weight, sagittal abdominal diameter, hypertension, serum total cholesterol, white blood cell count, history of coronary heart disease, diabetes, COPD, stroke, intermittent claudication, estimated GFR, HRT (women)
Wong DR et al, 2007, USA	Health Professionals Follow-up Study	1986-2002, 16 years follow-up	39352 men, age 40-75 years: 376 AAA cases	Smoking status	Never<br>Former, quit ≥10 yrs<br>Former, quit <10 yrs<br>Current, 1-4 cig/d<br>Current, 5-14 cig/d<br>Current 15-24 cig/d<br>Current, ≥25 cig/d	1.0<br>2.5 (1.8-3.6)<br>6.5 (4.5-9.3)<br>1.8 (0.4-7.4)<br>5.9 (3.0-11.4)<br>14.2 (9.4-21.5)<br>15.2 (9.9-23.3)	Age, hypertension, diabetes, hypercholesterolemia, BMI, physical activity
Lederle FA et al, 2008, USA	Women's Health Initiative	1993-1998 - 2004-2005, 7.8 years follow-up	161808 women, age 50-79 years: 184 AAA cases	Smoking status<br><br><br>Pack-years	Never<br>Ever<br>Current<br>Per 5 pack-years	1.00<br>1.94 (1.16-3.24)<br>8.73 (5.04-15.12)<br>1.11 (1.08-1.14)	Age, ethnicity/race, height, weight, hypertension, drugs for high cholesterol, coronary artery disease, cerebrovascular disease, DM, COPD, HT, peripheral artery disease, venous thromboembolism, non-skin cancer, previous aortic aneurysm, alcohol
Lawlor DA et al, 2008, South Korea	Korean Medical Insurance Corporation	1992 - 2001, 10 years follow-up	648346 men, age 30-64 years: 269 AA cases	Smoking status	Never<br>Former<br>Current, <10 cig/d<br>Current, 10-19 cig/d<br>Current, ≥20	1.00<br>1.23 (0.81-1.63)<br>1.63 (1.05-2.55)<br>1.78 (1.18-2.70)<br>1.36 (0.85-2.18)	Age, height, blood pressure, BMI, cholesterol, hyperglycemia, alcohol, regular exercise, income, area of residence

Forsdahl SH et al, 2009, Norway	The Tromsø Study	1994/1995 - 2001, 7 years follow-up	2035 men and 2310 women, age 25-82 years: 119 AAA cases	Smoking status<br><br><br><br><br><br><br>Duration	Never<br>Stopped ≥20 years<br>Stopped 10-19 years<br>Stopped <10 years<br>Current, <10 cig/d<br>Current, 10-19 cig/d<br>Current, ≥20 cig/d<br>Never<br>Smoked <20 years<br>Smoked 20-29<br>Smoked ≥30	1.00<br>1.26 (0.54-2.96)<br>2.90 (1.25-6.72)<br>2.88 (1.23-6.75)<br>6.19 (2.86-13.38)<br>9.78 (4.89-19.58)<br>13.72 (6.12-30.78)<br>1.00<br>0.44 (0.12-1.58)<br>2.56 (1.17-5.58)<br>7.01 (3.73-13.16)	Age, sex, total cholesterol, serum HDL-cholesterol, statin use, hypertension
Pirie K et al, 2012, United Kingdom	Million Women's Health Study	1996-2001 – 2011, 12 years follow-up	1180652 women, age 50-69 years: 330/164 AAA deaths	Smoking status<br><br><br>Cigarettes per day	Never<br>Former<br>Current<br><10 cig/d<br>10-19<br>≥20	1.00<br>-<br>6.32 (5.17-7.71)<br>3.87<br>7.18<br>8.09	Age, geographical region, BMI, SES, alcohol, strenuous physical activity, height, OC use, menopausal status, menopausal hormone therapy use
Sode BF et al, 2013, Denmark	Copenhagen City Heart study	1976-1978 - 2010, 23 years follow-up	15072 men and women, age 20-≥80 years: 335 AAA cases	Smoking status	Never<br>Former<br>Current<br>0 g/d<br>1-20 g/d<br>>20 g/d	1.0<br>1.5 (0.8-2.7)<br>3.5 (2.2-5.8)<br>1.0<br>3.1 (1.9-5.0)<br>3.4 (1.9-5.8)	Age, sex, SBP, cholesterol, alcohol, lipid lowering medication, anti-hypertensive medication
Sode BF et al, 2013, Denmark	Copenhagen General Population Study	2003 - 2010, 4 years follow-up	56211 men and women, age 20-≥80 years: 169 AAA cases	Smoking status	Never<br>Former<br>Current<br>0 g/d<br>1-20 g/d<br>>20 g/d	1.0<br>1.9 (1.2-3.0)<br>3.8 (2.4-6.1)<br>1.0<br>2.1 (1.3-3.2)<br>3.4 (2.1-5.4)	Age, sex, SBP, cholesterol, alcohol, lipid lowering medication, anti-hypertensive medication
Stackelberg O et al, 2014, Sweden	Swedish Mammography Cohort Study	1998-2011, 12.7 years follow-up	35550 women, age 46-84 years: 199 AAA cases	Smoking status	Never<br>Former, <20 yrs quit<br>Former, ≥20 yrs quit<br>Current, <20 pack-yrs<br>Current, ≥20 pack-yrs	1.00<br>4.63 (3.04-7.06)<br>0.82 (0.35-1.92)<br>7.01 (4.63-10.62)<br>10.97 (7.41-16.26)	Age, education, waist circumference, alcohol, fruit, diabetes, hypertension, hypercholesterolemia, cardiovascular disease

Stackelberg O et al, 2014, Sweden	Cohort of Swedish Men	1998-2011, 12.7 years follow-up	42596 men, age 46-84 years: 958 AAA cases	Smoking status	Never<br>Former, <20 yrs quit<br>Former, ≥20 yrs quit<br>Current, <20 pack-yrs<br>Current, ≥20 pack-yrs	1.00<br>3.77 (3.08-4.63)<br>1.61 (1.27-2.03)<br>3.06 (2.37-3.95)<br>6.55 (5.36-7.99)	Age, education, waist circumference, alcohol, fruit, diabetes, hypertension, hypercholesterolemia, cardiovascular disease
Svensjo S et al, 2014, Sweden	Uppsala men born 1941-1942	2006-2007 - 2011-2012, 5 years follow-up	2059 men, age 65 years: 36 AAA cases	Smoking status	Never<br>Current	1.00<br>2.78 (1.38-5.57)	Coronary disease, claudication, sub-aneurysmal aorta at 65 years, infrarenal aortic diameter at 65 years
Howard DPJ et al, 2015, United Kingdom	The Oxford Vascular Study	2002-2014, 12 years follow-up	92728 men and women, age 45-≥85 years: 103 AAA cases	Smoking status, men<br><br><br>Smoking status, women	Never<br>Ever<br>Current<br>Never<br>Ever<br>Current	1.00<br>1.60 (1.44-1.77)<br>2.30 (1.72-3.06)<br>1.00<br>1.35 (1.00-1.82)<br>1.63 (0.80-3.33)	Age
Jahangir E et al, 2015, USA	Southern Community Cohort Study	1999-2012, 4.94 years follow-up	18782 men and women, age ≥65 years: 281 AAA cases	Smoking status, all<br><br><br>Smoking status, men<br><br><br>Smoking status, women	Never<br>Former<br>Current<br>Never<br>Former<br>Current<br>Never<br>Former<br>Current	1.00<br>1.91 (1.27-2.87)<br>5.55 (3.67-8.40)<br>1.00<br>1.10 (0.65-1.86)<br>3.40 (1.96-5.90)<br>1.00<br>3.40 (1.83-6.31)<br>9.17 (4.95-17.0)	Age, sex, ethnicity, education, BMI, MI/CABG, high blood pressure, high cholesterol, diabetes
Pujades-Rodriguez M et al, 2015, United Kingdom	The Clinical Practice Research Datalink	1997-2010, 5.5 years follow-up	1937360 men and women, age ≥30 years: 3135 AAA cases	Smoking status, all<br><br><br><br><br>Smoking status, men<br><br>Smoking status, women	Never<br>Former, <2 yrs quit<br>Former, 2-9 yrs quit<br>Former, ≥10 yrs quit<br>Current<br>Never<br>Current<br>Never<br>Current	1.00<br>4.31 (2.34-7.94)<br>3.98 (2.67-5.94)<br>1.30 (1.02-1.65)<br>5.18 (4.61-5.82)<br>1.00<br>5.48 (4.78-6.29)<br>1.00<br>4.48 (3.59-5.58)	Age
Tang W et al, 2016, USA	Atherosclerosis Risk in Communities Study	1987-1989 - 2013, 22.5 years follow-up	15792 men and women, age 45-64 years: 590 AAA cases	Smoking status, all<br><br><br>Pack-years	Never<br>Former<br>Current<br>None<br>0.1-15.2 pack-years	1.00<br>2.34 (1.11-4.95)<br>6.86 (3.18-14.8)<br>1.00<br>1.46 (0.56-3.78)	Age, sex, race, height, alcohol, triglycerides, total cholesterol

					15.3-33.9<br>34.0-243	3.59 (1.64-7.84)<br>5.91 (2.76-12.6)	
				Smoking status, longitudinal smoking status, clinical AAA	Never<br>Quitter before visit 1<br>Recent quitter<br>Current smoker	1.00<br>1.83 (1.19-2.81)<br>3.50 (1.53-8.04)<br>6.41 (3.67-11.2)	
				Smoking status, longitudinal smoking status, asymptomatic AAA	Never<br>Quitter before visit 1<br>Recent quitter<br>Current smoker	1.00<br>1.34 (0.60-2.99)<br>2.66 (0.65-10.9)<br>3.93 (1.44-10.7)	
Kihara T et al, 2017, Japan	Japan Collaborative Cohort Study	1988-1990 - 2009, 19 years follow-up	48677 men and women, age 40-79 years: 75 AA deaths	Active/passive smoking	Never/low<br>Never/intermediate<br>Never/high<br>Former<br>Current	1.00<br>1.12 (0.34-3.67)<br>1.89 (0.50-7.17)<br>1.52 (0.47-4.95)<br>4.58 (1.59-13.19)	Age, sex, BMI, hypertension, alcohol, perceived mental stress, walking, age of completed education, job status, region

BMI =Body mass index, CABG=coronary artery bypass grafting, COPD=chronic obstructive pulmonary disease, DBP=diastolic blood pressure, DM=diabetes mellitus, GFR=glomerular filtration rate, HDL=high-density lipoprotein, HRT=hormone replacement therapy, HT=hormone therapy, MI=myocardial infarction, NA= not available, OC use=oral contraceptive use, SBP=systolic blood pressure, SES=socioeconomic status, TG=triglycerides, yrs=years

Supplementary Table 3. Cigarettes per day and abdominal aortic aneurysm

Cigarettes/day	RR (95% CI)
0	1.00
5	1.99 (1.67-2.37)
10	3.52 (2.58-4.80)
15	4.99 (3.41-7.29)
20	5.81 (3.92-8.60)
25	5.91 (4.04-8.64)
30	5.61 (3.88-8.11)
$p_{nonlinearity}$	<0.0001

Supplementary Table 4. Pack-years of smoking and abdominal aortic aneurysm

Years since quitting	RR (95% CI)
0	1.00
5	2.01 (1.38-2.92)
10	3.60 (1.87-6.94)
15	5.34 (2.46-11.63)
20	6.61 (3.11-14.11)
25	7.17 (3.82-13.45)
30	7.08 (4.55-11.01)
$p_{nonlinearity}$	0.02

Supplementary Table 5. Years since quitting smoking and abdominal aortic aneurysm

Years since quitting	RR (95% CI)
0 (current smokers)	1.00
5	0.68 (0.55-0.85)
10	0.46 (0.33-0.65)
15	0.31 (0.20-0.48)
20	0.20 (0.11-0.36)
25	0.13 (0.06-0.30)
29	0.10 (0.03-0.27)
$p_{nonlinearity}$	0.85

Supplementary Figure 1. Funnel plot of current smoking and abdominal aortic aneurysm

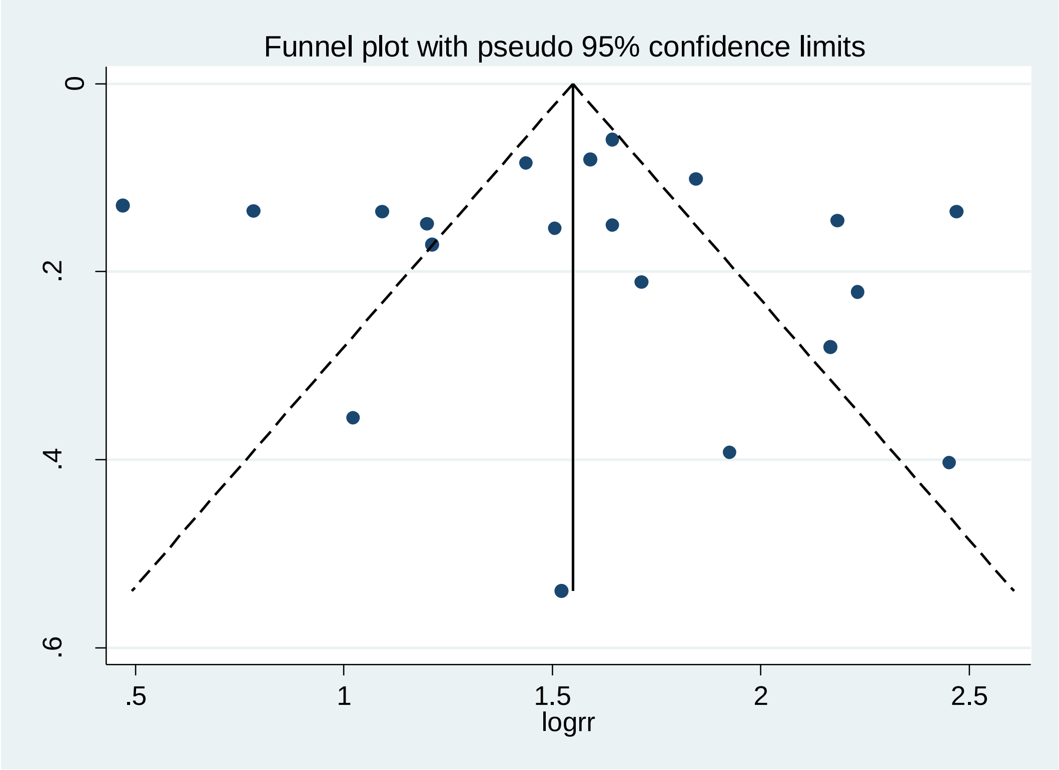

Supplementary Figure 2. Funnel plot of former smoking and abdominal aortic aneurysm

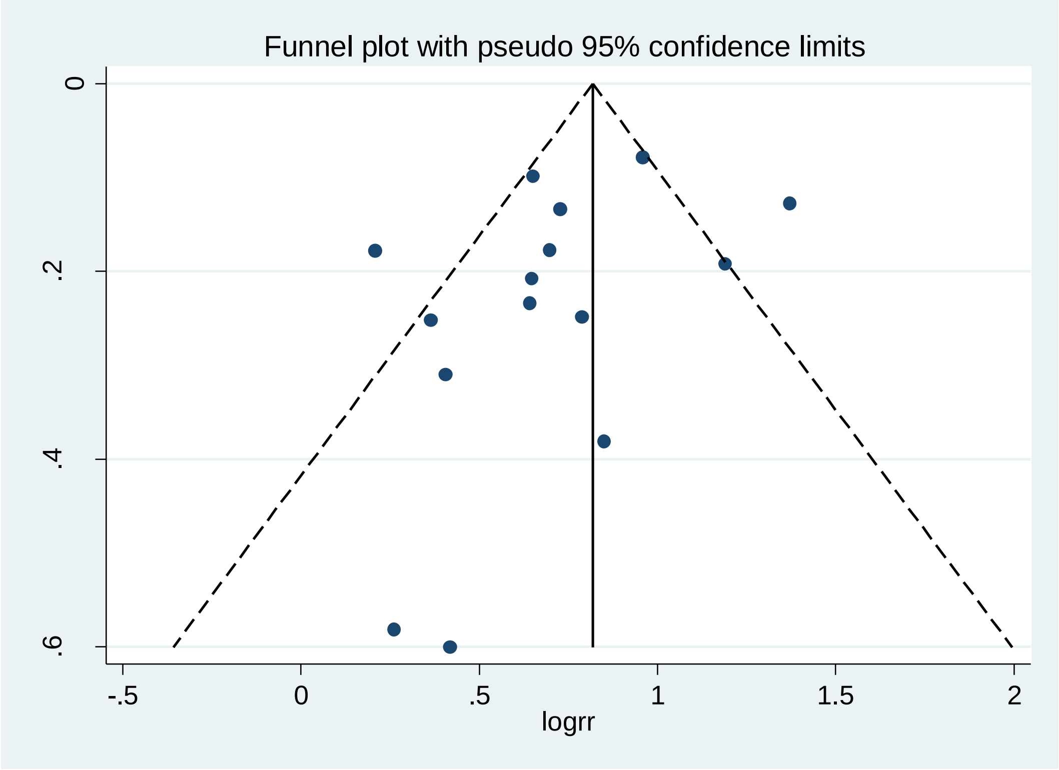

Supplementary Figure 3. Funnel plot of ever smoking and abdominal aortic aneurysm

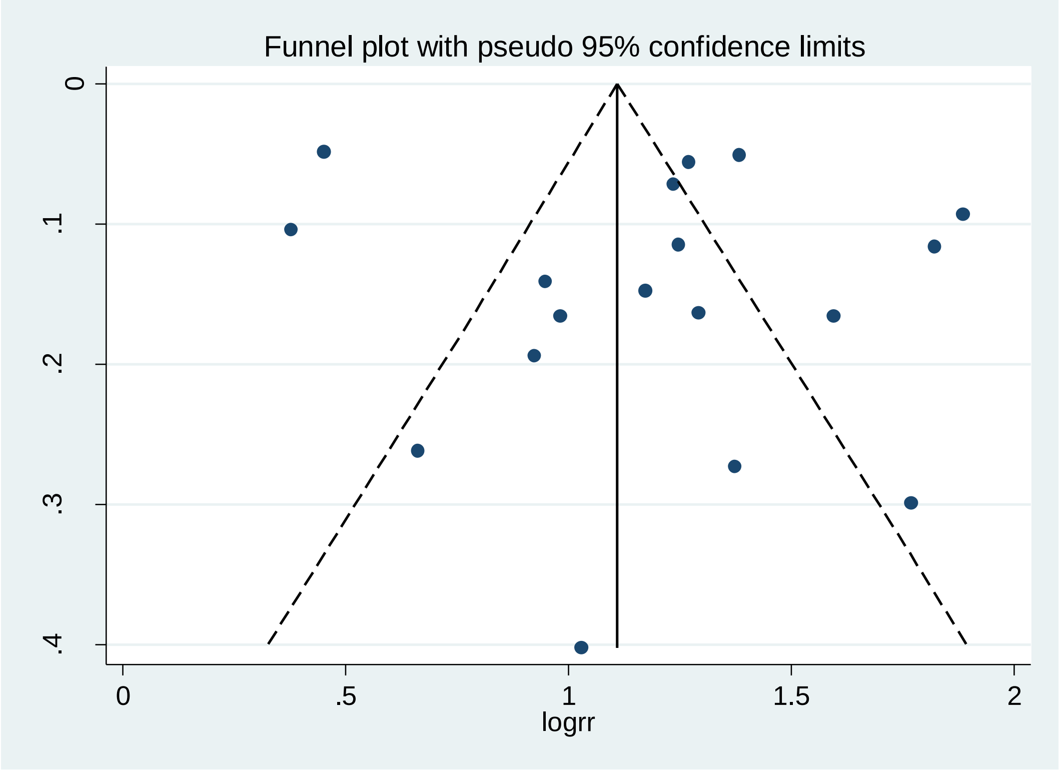

Supplementary Figure 4. Influence analysis of current smoking and abdominal aortic aneurysms

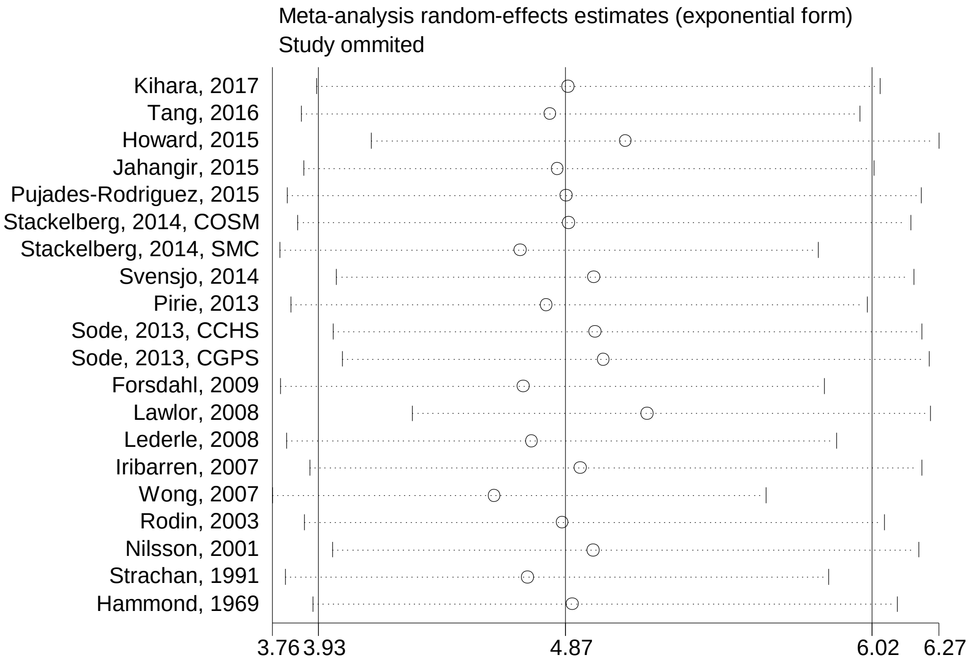

Study omitted	e^coef.	[95% Conf.	Interval]
Kihara, 2017	4.8732638	3.9257429	6.0494795
Tang, 2016	4.8072619	3.8688896	5.9732304
Howard, 2015	5.0910692	4.132822	6.2714982
Jahangir, 2015	4.833982	3.8768985	6.0273385
Pujades-Rodriguez, 2015	4.8656816	3.8153093	6.2052269
Stackelberg, 2014, COSM	4.8754168	3.8557346	6.164763
Stackelberg, 2014, SMC	4.6936235	3.7873847	5.8167057
Svensjo, 2014	4.9707479	4.0009637	6.1755958
Pirie, 2013	4.7928729	3.828316	6.0004535
Sode, 2013, CCHS	4.9758964	3.9890194	6.2069249
Sode, 2013, CGPS	5.0079312	4.0224895	6.2347898
Forsdahl, 2009	4.7043858	3.7901385	5.8391647
Lawlor, 2008	5.1716447	4.2874017	6.2382565
Lederle, 2008	4.7375731	3.8134484	5.885644
Iribarren, 2007	4.9199843	3.8997586	6.2071137
Wong, 2007	4.5970559	3.7602458	5.620091
Rodin, 2003	4.8517132	3.8803513	6.0662341
Nilsson, 2001	4.9686847	3.9850729	6.195076
Strachan, 1991	4.7217889	3.8078179	5.8551359
Hammond, 1969	4.8904333	3.9115434	6.1142974
Combined	4.8652094	3.9327087	6.0188192

Supplementary Figure 5. Influence analysis of former smoking and abdominal aortic aneurysms

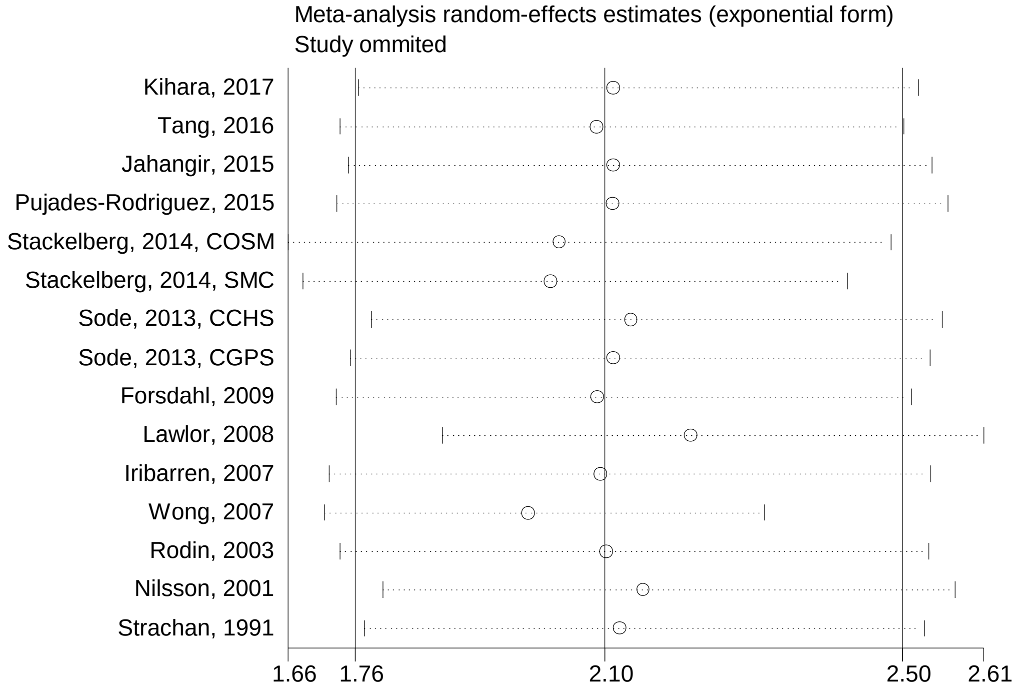

Study omitted	e^coef.	[95% Conf.	Interval]
Kihara, 2017	2.1073267	1.7600673	2.5231004
Tang, 2016	2.0838819	1.7350233	2.5028849
Jahangir, 2015	2.1067533	1.7463551	2.541528
Pujades-Rodriguez, 2015	2.1062405	1.7307212	2.5632374
Stackelberg, 2014, COSM	2.0337012	1.6642301	2.4851975
Stackelberg, 2014, SMC	2.0216346	1.684405	2.4263797
Sode, 2013, CCHS	2.1313329	1.7775302	2.5555568
Sode, 2013, CGPS	2.1065922	1.7482027	2.5384529
Forsdahl, 2009	2.0850933	1.7297008	2.5135067
Lawlor, 2008	2.2126584	1.8744295	2.6119189
Iribarren, 2007	2.089792	1.7198766	2.5392699
Wong, 2007	1.9909173	1.7138845	2.3127298
Rodin, 2003	2.0978281	1.7346416	2.537056
Nilsson, 2001	2.1477613	1.7929132	2.5728402
Strachan, 1991	2.1154137	1.768082	2.5309768
Combined	2.0954087	1.7555035	2.5011273

Supplementary Figure 6. Influence analysis of ever smoking and abdominal aortic aneurysms

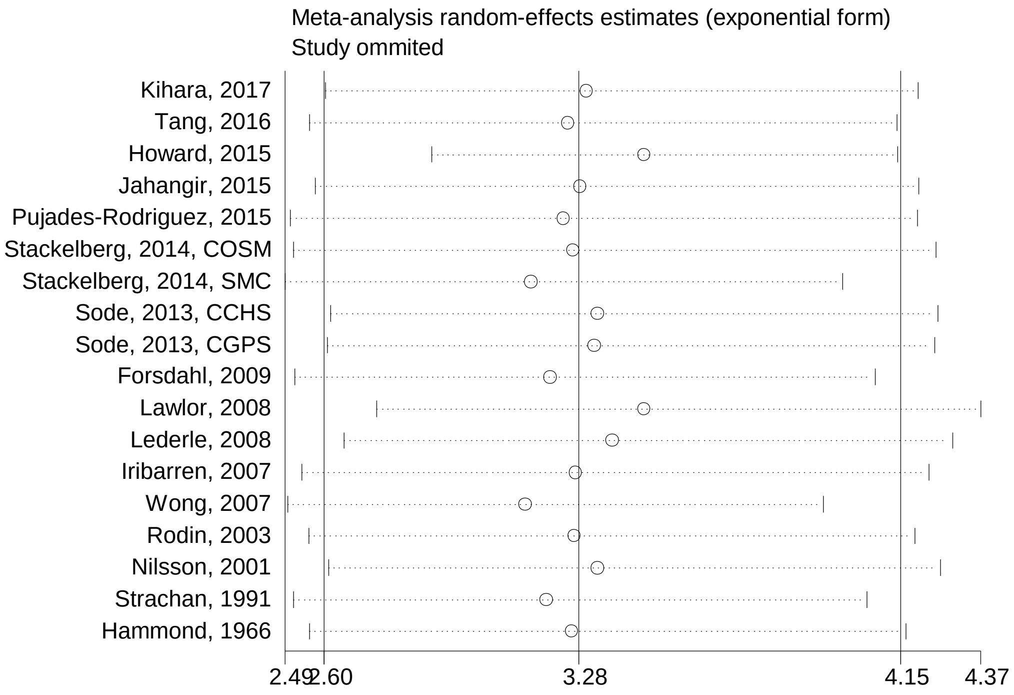

Study omitted	e^coef.	[95% Conf.	Interval]
Kihara, 2017	3.3043883	2.6013534	4.1974235
Tang, 2016	3.2544129	2.5579298	4.1405377
Howard, 2015	3.4589932	2.8881483	4.1426659
Jahangir, 2015	3.2877905	2.574275	4.1990719
Pujades-Rodriguez, 2015	3.2434082	2.5076098	4.1951094
Stackelberg, 2014, COSM	3.2678764	2.515053	4.2460403
Stackelberg, 2014, SMC	3.1553001	2.4933014	3.993067
Sode, 2013, CCHS	3.3343072	2.6160326	4.2497964
Sode, 2013, CGPS	3.3249993	2.6059425	4.242465
Forsdahl, 2009	3.2062051	2.518661	4.0814352
Lawlor, 2008	3.4588571	2.7400355	4.3662543
Lederle, 2008	3.3731148	2.6521847	4.2900119
Iribarren, 2007	3.2749832	2.5377183	4.2264404
Wong, 2007	3.1386313	2.4992638	3.9415638
Rodin, 2003	3.2726018	2.5570443	4.1883998
Nilsson, 2001	3.3333077	2.6098104	4.2573748
Strachan, 1991	3.1956635	2.5156772	4.0594501
Hammond, 1966	3.2644033	2.5586562	4.1648149
Combined	3.2843279	2.5988565	4.1505984

Moose checklist_smoking and abdominal aortic aneurysm

Reporting of background should include	Page
Problem definition	4,5
Hypothesis statement	4,5
Description of study outcome(s)	4,5,6
Type of exposure or intervention used	4,5,6
Type of study designs used	4,5,6
Study population	4,5
Reporting of search strategy should include	
Qualifications of searchers (eg, librarians and investigators)	6
Search strategy, including time period included in the synthesis and keywords	Supplementary text
Effort to include all available studies, including contact with authors	No contact with authors
Databases and registries searched	6
Search software used, name and version, including special features used (eg, explosion)	6
Use of hand searching (eg, reference lists of obtained articles)	6
List of citations located and those excluded, including justification	9, Supplementary Table 1
Method of addressing articles published in languages other than English	No non-english articles were identified
Method of handling abstracts and unpublished studies	Not included
Description of any contact with authors	No contact with authors
Reporting of methods should include	
Description of relevance or appropriateness of studies assembled for assessing the hypothesisto be tested	6,7, Supplementary Table 2
Rationale for the selection and coding of data (eg, sound clinical principles or convenience)	6

Documentation of how data were classified and coded (eg, multiple raters, blinding, andinterrater reliability)	6
Assessment of confounding (eg, comparability of cases and controls in studies whereappropriate)	7,8, Supplementary Table 2
Assessment of study quality, including blinding of quality assessors; stratification or regressionon possible predictors of study results	7
Assessment of heterogeneity	7
Description of statistical methods (eg, complete description of fixed or random effects models,justification of whether the chosen models account for predictors of study results,dose-response models, or cumulative meta-analysis) in sufficient detail to be replicated	6-8
Provision of appropriate tables and graphics	Table 1, Figure 1-5, Supplementary Table 1-5, Supplementary Figure 1-6
Reporting of results should include	
Graphic summarizing individual study estimates and overall estimate	8-10, Figure 2-5
Table giving descriptive information for each study included	Supplementary Table 2
Results of sensitivity testing (eg, subgroup analysis)	10, Table 1
Indication of statistical uncertainty of findings	8-10, Figure 2-5, Supplementary Table 2
Reporting of discussion should include	
Quantitative assessment of bias (eg, publication bias)	11-12
Justification for exclusion (eg, exclusion of non–English-language citations)	Non-English studies not identified
Assessment of quality of included studies	13
Reporting of conclusions should include	
Consideration of alternative explanations for observed results	11-13

Generalization of the conclusions (ie, appropriate for the data presented and within the domainof the literature review)	12-13
Guidelines for future research	13
Disclosure of funding source	14
